# Biomimetic peroxidase MOF-Fe promotes bone defect repair by inhibiting TfR2 and activating the BMP2 pathway

**DOI:** 10.1186/s13062-024-00473-2

**Published:** 2024-04-23

**Authors:** Yaxin Xue, Wei Xu, Danyang Zhao, Zijing Du, Hao Jiang, Hao Lv, Dong Zhang, Zhencheng Yu, Yi Cao, Dong Han

**Affiliations:** 1 Department of Plastic and Reconstructive Surgery, Shanghai Ninth People’s Hospital, Shanghai Jiao Tong University School of Medicine, Shanghai Ninth People’s Hospital, 639 Zhizaoju Road 200011, Shanghai, People’s Republic of China; 2 Engineering Research Center for Biomedical Materials of Ministry of Education, East China University of Science and Technology, 200237 Shanghai, P. R. China

**Keywords:** Bone mesenchymal stem cells, Bone defect repair, Iron metabolism, Reactive oxygen species

## Abstract

**Background:**

Large bone defects pose a clinical treatment challenge; inhibiting transferrin receptor 2 (TfR2), which is involved in iron metabolism, can promote osteogenesis. Iron-based metal-organic frameworks (MOF-Fe) particles not only inhibit TfR2 but also serve as biomimetic catalysts to remove hydrogen peroxide in reactive oxygen species (ROS); excess ROS can disrupt the normal functions of osteoblasts, thereby hindering bone regeneration. This study explored the potential effects of MOF-Fe in increasing osteogenic activity and clearing ROS.

**Methods:**

In vitro experiments were performed to investigate the osteogenic effects of MOF-Fe particles and assess their impact on cellular ROS levels. To further validate the role of MOF-Fe in promoting bone defect repair, we injected MOF-Fe suspensions into the femoral defects of SD rats and implanted MOF-Fe-containing hydrogel scaffolds in rabbit cranial defect models and observed their effects on bone healing.

**Results:**

In vitro, the presence of MOF-Fe significantly increased the expression levels of osteogenesis-related genes and proteins compared to those in the control group. Additionally, compared to those in the untreated control group, the cells treated with MOF-Fe exhibited a significantly increased ability to remove hydrogen peroxide from ROS and generate oxygen and water within the physiological pH range. In vivo experiments further confirmed the positive effect of MOF-Fe in promoting bone defect repair.

**Conclusion:**

This study supports the application of MOF-Fe as an agent for bone regeneration, particularly for mitigating ROS and activating the bone morphogenetic protein (BMP) pathway, demonstrating its potential value.

**Supplementary Information:**

The online version contains supplementary material available at 10.1186/s13062-024-00473-2.

## Introduction

Healing of large bone defects is a challenge in clinical treatment. Many studies have explored the therapeutic potential of iron-containing particles in promoting bone repair [1–3], with most reports focusing on the application of iron oxide nanoparticles (IONPs, Fe_3_O_4_/γ-Fe_2_O_3_). For example, Yang Xia et al. promoted the osteogenic differentiation of human dental pulp stem cells by incorporating IONPs into calcium phosphate cement scaffolds. After IONPs were incorporated into the scaffolds, the expression of Alkaline Phosphatase (ALP) and osteogenesis-related genes increased by approximately threefold, and the efficacy of osteogenesis improved. An increase in the iron ion concentration in iron metabolism further inhibited the expression of transferrin receptor 2 (TfR2). Furthermore, M. Rauner U et al. reported that TfR2, which is involved in iron metabolism, can limit bone formation by regulating Bone Morphogenetic Protein (BMP) signalling [4]. After knocking out the TfR2 gene in mice, these researchers found an increase in mineralization and bone mass. Iron-containing particles can increase BMP-mediated osteogenesis by inhibiting TfR2 expression through iron. However, the current application of IONPs is limited because IONPs are inorganic materials with poor biocompatibility. As an iron-containing particle, the iron-based metal-organic frameworks (MOF-Fe) comprises organic ligands and iron ions. Compared with that of IONPs, the main structure of MOF-Fe is organic, making MOF-Fe more biocompatible.

The healing process of bone defects is influenced by multiple factors, and the role of reactive oxygen species (ROS) in bone repair has attracted widespread attention [5–7]. Excess ROS disrupt the balance of bone tissue by inhibiting osteoblast activity and promoting osteoclast formation, resulting in decreased bone mass and density [8–10]. In the categories of ROS, specific entities include superoxide anion (O_2_^•–^), hydrogen peroxide (H_2_O_2_), and hydroxyl radicals (•OH) [5]. During cellular metabolism, hydrogen peroxide, a common byproduct, typically occurs at higher concentrations than other ROS. Additionally, a certain degree of interconversion exists between hydrogen peroxide and other ROS. For example, excess hydroxyl radicals can be generated from hydrogen peroxide through the Fenton reaction. Concurrently, within cells, superoxide dismutase (SOD) catalyses the transformation of superoxide anions into hydrogen peroxide. Based on these mechanisms, developing hydrogen peroxide scavengers may provide a viable approach to exploring effective materials for ROS removal.

MOF-Fe are renowned for their unique unsaturated metal centres (UMCs) [11–13]. This distinctive feature gives MOF-Fe a high energy density, making it a potential catalyst that effectively mimics the function of natural peroxidases by catalysing the decomposition of hydrogen peroxide. As such, MOF-Fe has widespread applications in peroxidase mimetics. In environmental applications, MOF-Fe catalyses wastewater treatment and ecological purification, facilitating the breakdown of harmful hydrogen peroxide and other organic pollutants and thus reducing pollution. In biomedicine, MOF-Fe is employed in biosensors to detect and monitor hydrogen peroxide levels within biological systems, which is crucial for disease diagnostics and physiological monitoring. For example, Wu et al. [14] developed the Fe_3_O_4_ magnetic catalyst coated with MOF-Fe for trace-level cholesterol detection, leveraging the additional iron catalytic sites of MOF-Fe to decompose H_2_O_2_. Liu et al. [15] fabricated a novel biosensor by growing porphyrin-based iron MOF (pFeMOF) crystals on glassy carbon electrodes. This modified electrode exhibited a low detection limit for hydrogen peroxide at 0.45 × 10^− 6^ M, demonstrating the effectiveness of pFeMOF in increasing hydrogen peroxide detection and highlighting the potential of MOF-Fe in catalysing hydrogen peroxide decomposition.

In this study, we explored the osteogenic potential of MOF-Fe and examined its effects on the proliferation, differentiation, and mineralization of bone marrow mesenchymal stem cells (BMSCs). By combining materials science with bone biology, we aim to contribute to the development of effective bone repair and regeneration strategies.

## Results

### Material characteristics

**Fig. 1:**
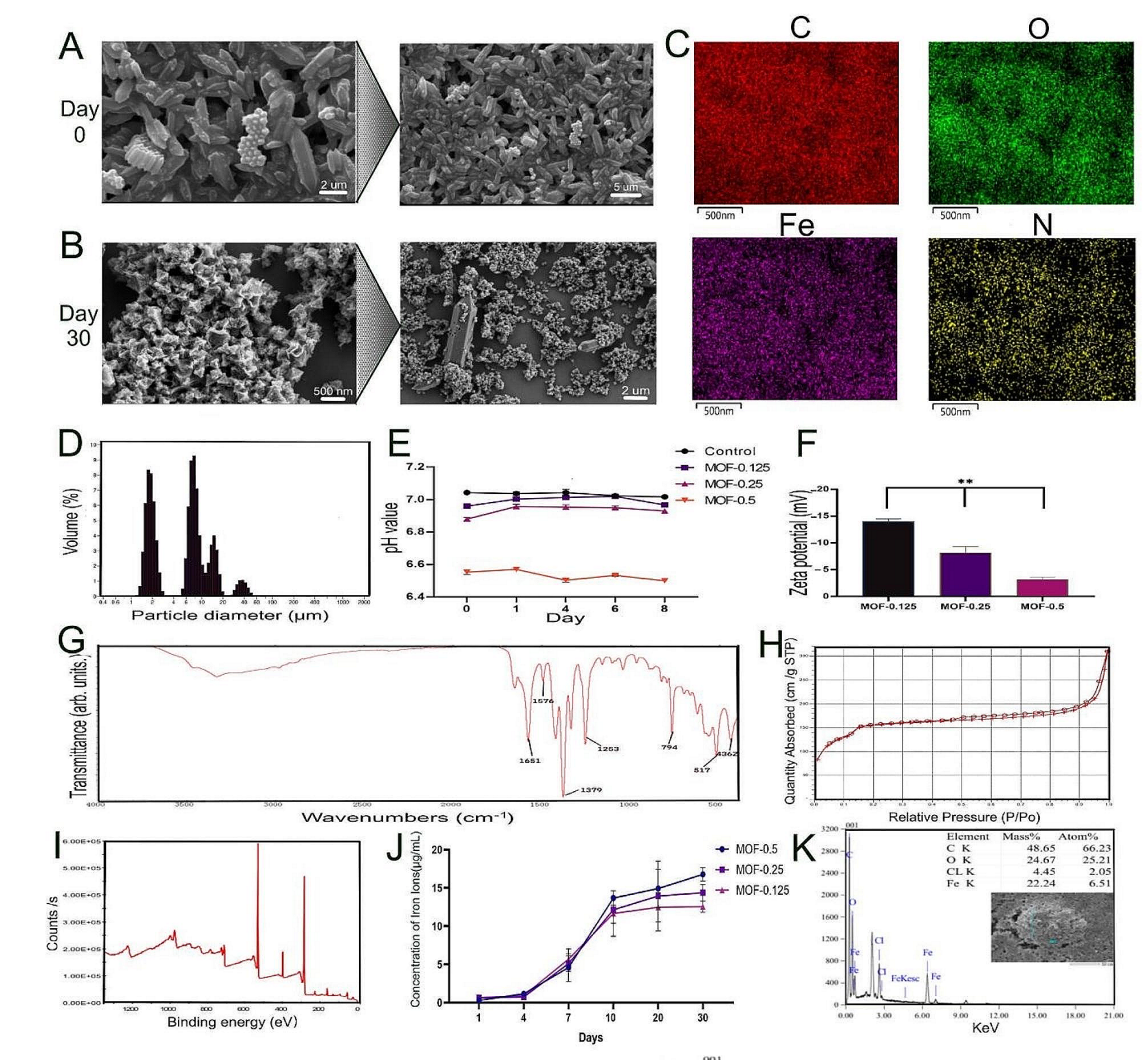
Detection and performance analysis of iron-based metal-organic frameworks (MOF-Fe). **A**: Scanning electron microscopy (SEM) image of MOF-Fe postsolvothermal synthesis, predominantly revealing polyhedral prism-like particles. **B**: SEM image of MOF-Fe after 30 days, showing degradation into smaller octahedra. **C**: Elemental mapping images of MOF-Fe nanoparticles, illustrating the distribution of carbon, oxygen, nitrogen, and iron within the MOF-Fe particles. **D**: The particle size distribution of the MOF-Fe particles shows an average cross-sectional diameter of 1.80 μm and a height of 11.50 μm. **E**: pH measurement curves of MOF-Fe suspensions at varying concentrations and in the control groups over different time points (zero days, one day, four days, six days, and eight days). The solvent is simulated body fluid. The pH was maintained within a physiological range of 6.50 to 7.40, demonstrating good stability. **F**: Absolute zeta potential measurements of MOF-Fe nanoparticles at various concentrations at 25 °C. **G**: Fourier transform infrared (FT-IR) spectroscopy. **H**: Pore size distribution of MOF-Fe nanoparticles, with a surface area of 553.48 m²/g, total pore volume of 0.32 cm³/g, micropore volume of 0.069 cm³/g, and average pore diameter of 4.10 nm. **I**: X-ray photoelectron spectroscopy (XPS) assessment results. **J**: Inductively coupled plasma optical emission spectroscopy (ICP‒OES) curves of free iron ions in different MOF-Fe suspension supernatants from the start of the experiment to Day 30. **K**: The elemental composition of the MOF-Fe particles was determined by energy dispersive X-ray spectroscopy (EDS), and the proportions of C, O, and Fe were 66.32%, 25.21%, and 6.51%, respectively. The data are presented as the means ± SDs (*n* = 3). *p < 0.05, **p < 0.01

Following solvothermal synthesis, the morphological features of the MOF-Fe particles were elucidated using scanning electron microscopy. Most of the MOF-Fe particles exhibited a polyhedral prism shape (Fig. 1A), with an average cross-sectional diameter of 1.80 μm and a prism height averaging 11.50 μm (Fig. 1D). For determination of their stability, the pH values of the MOF-0.125, MOF-0.25, and MOF-0.5 groups and the control group were measured on Days 0, 1, 4, 6, and 8. The results revealed a dose-dependent variation in pH. Specifically, the pH values for the MOF-Fe suspensions of 0.5 and 0.25 mg/mL oscillated between 6.50 and 6.57 and between 6.88 and 6.96, respectively, over 0–8 days. For the 0.125 mg/mL MOF-Fe suspension, the pH approached 7.00, with values of 6.96–7.02. Notably, all group pH values remained within the physiological range (6.50 ∼ 7.40) and demonstrated relative stability; time exhibited no significant influence on pH variation (Fig. 1E).

After sonication treatment of MOF-Fe nanoparticles at various concentrations, zeta potentials were gauged at a constant temperature of 25 °C (Fig. 1F). The results showed that the absolute zeta potentials for the MOF-0.125, MOF-0.25, and MOF-0.5 groups were 14.03 ± 0.38, 8.16 ± 0.95, and 3.19 ± 0.34, respectively. An increasing trend in the concentration of MOF-Fe nanoparticles correlated with a decreasing trend in the absolute zeta potential. This pattern suggests that nanoparticles in the MOF-0.5 group possess a relatively low surface charge, thus potentially reducing intercharge repulsion and facilitating interactions with charged molecules or ions. Elemental mapping analysis was conducted to further elucidate the structural characteristics of the MOF-Fe nanoparticles (Fig. 1C). After appropriately fixing the sample, we generated mapping images for carbon, oxygen, nitrogen, and iron during this process. This method revealed the distribution of each element within the MOF-Fe nanoparticles.

Fourier transform infrared spectroscopy (FT-IR) analysis revealed a peak corresponding to the -N-H stretching vibration between 3200 and 3500 cm^− 1^, albeit faintly. The bending vibration of the -N-H bond is discernible at 1650 cm^− 1^, while the peak at 1253 cm^− 1^ is attributed to the presence of aromatic amines. Furthermore, dual mounts observed at 1576 and 1379 cm^− 1^ corroborate the prominence of coordinating carboxylate in the MOF-Fe constitution, suggesting the presence of dicarboxylate linkers. The peak at 794 cm^− 1^ is associated with the C-H bending vibration of the aromatic ring, whereas characteristic peaks for the Fe-O bond appear at 517 and 436 cm^− 1^ (Fig. 1G).

With Brunauer‒Emmett‒Teller (BET) pore size analysis, the porous structure and surface characteristics of the MOF-Fe nanoparticles were determined by employing nitrogen as the adsorbing gas, and the results were analysed at a 77 K liquid nitrogen temperature (Fig. 1H). The MOF-Fe nanoparticles had a BET surface area of 553.48 m²/g. Concurrently, the pore volume was 0.32 cm³/g, the microporous volume was 0.069 cm³/g, and the average pore diameter was 4.10 nm.

Energy-dispersive X-ray spectroscopy (EDS) was used to meticulously discern the elemental composition of the MOF-Fe particles (Fig. 1K). This analysis revealed that the C, O, and Fe atomic ratios were 66.32%, 25.21%, and 6.51%, respectively. To further confirm the presence of N atoms, we used X-ray photoelectron spectroscopy (XPS) (Fig. 1I), and the N characteristic peak near 400 eV was successfully detected. Based on the detection area, the proportions of C, O, Fe, and N were 64.80%, 23.61%, 3.14%, and 8.45%, respectively, consistent with the EDS data.

Supernatants from various concentrations of MOF suspensions were periodically extracted from the beginning of the experiment to Day 30 and analysed via inductively coupled plasma optical emission spectroscopy (ICP‒OES) to further elucidate the behaviour of free iron ions in the MOF suspension (Fig. 1J). The results revealed that from inception to Day 10, the iron concentrations in all MOF-Fe suspensions progressively increased. However, iron release did not significantly increase from Day 10 to Day 30.

### Identification of the trilineage differentiation of BMSCs

**Fig. 2:**
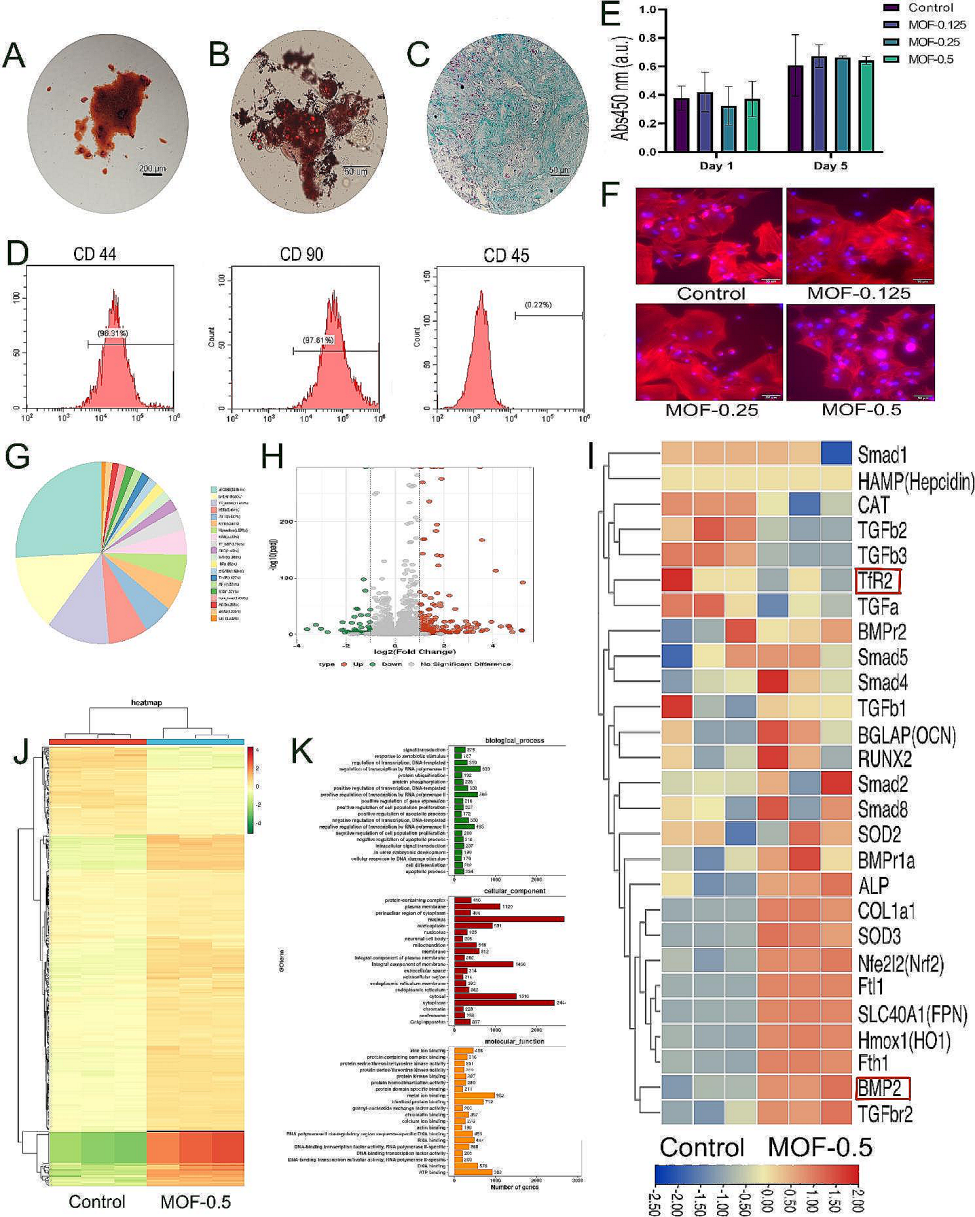
Identification of Bone Marrow Stromal Cells (BMSCs) and the Impact of MOF-Fe Nanoparticles on Cell Viability. **A**-**C**. Tri-lineage differentiation demonstrates that the extracted cells possess stem cell characteristics. **D**. Flow cytometry analysis revealed a positive CD44 expression rate of 96.31%, a positive CD90 expression rate of 97.61%, and a positive CD45 expression rate of only 0.22%. **E**. Cell Counting Kit-8 (CCK-8) assays for cell viability demonstrating the nontoxic effect of MOF nanoparticle filtrates on BMSCs. **F**. No morphological changes in the cells were observed after cocultivation with the MOFs. **G**. The composition of thetranscription factor family, with the most significant sector representing the C2H2-type transcription factor. **H**. Differential gene expression between the control and MOF-0.5 groups revealed 285 genes, 227 of which were upregulated and 58 of which were downregulated. **I**. A heatmap was constructed to visualize the expression of specific genes, revealing a decrease in the expression of the iron metabolism-related gene Transferrin Receptor 2 (TfR2) and an increase in the expression of Bone Morphogenetic Protein 2 (BMP2) and its downstream pathway genes, including Runt-Related Transcription Factor 2 (RUNX2), Collagen Type I Alpha 1 Chain (COL1A1), and Alkaline Phosphatase (ALP). **J**. The heatmap compares the expression levels of all genes between the two sample groups (represented by different colours), with a gradient from red (high expression) to green (low expression) indicating the level of gene expression change. **K**. Bar chart of Gene Ontology (GO) terms illustrating the distribution of genes in three main categories, biological processes, cellular components, and molecular functions, providing an intuitive understanding of the breadth of gene function distribution. Each bar represents a GO term, with the length indicating the number of genes in that term. The data are presented as the means ± SDs (*n* = 3). *p < 0.05, **p < 0.01

BMSCs exhibited pluripotent differentiation upon culture in specific induction media (Fig. 2A-C). Moreover, phenotypic analyses were performed on rat BMSCs from passages 3 to 5 using flow cytometry (Fig. 2D). The findings revealed pronounced expression of CD44 and CD90, with positivity rates of 96.31% and 97.61%, respectively. In contrast, CD45 displayed only 0.22% positivity, which was markedly inferior to that of CD44 and CD90, consistent with the characteristic phenotype of mesenchymal stem cells.

### Assessment of cellular viability

The concentrations of the experimental groups were set at 0.125 mg/mL, 0.25 mg/mL, and 0.5 mg/mL, respectively, labelled as MOF-0.125, MOF-0.25, and MOF-0.5 referring to the optimal osteogenic concentration of iron-containing particles in related literature [3].

Data from the Cell Counting Kit-8 (CCK-8) assay revealed that MOF nanoparticle leachates did not significantly affect BMSCs at the concentrations evaluated (Fig. 2E). Subsequent fluorescence staining experiments employing TRITC-phalloidin and DAPI were used to investigate the influence of internalized MOF particles on cell adhesion morphology. The results confirmed that the morphology of MOF-labelled cells mirrored that of unmarked cells, with all BMSC groups uniformly displaying flat, multicellular overlapping features exhibiting optimal peripheral extension (Fig. 2F).

The pH range of the culture medium used for cell culture was determined to be neutral using pH test strips. After the addition of MOF-0.125, MOF-0.25, and MOF-0.5 to the culture medium, respectively, the colour change of the pH test strips was minimal, indicating a neutral pH range (Supporting Information Fig. 2).

### Transcriptomic analysis of second-generation BMSCs post-cocultivation with MOF-Fe

TransDecoder identified reliable coding sequences (CDSs) in newly discovered transcripts. Given the open reading frame (ORF) length and log-likelihood score, the analysis revealed that most of the sequences were less than 2500 nucleotides post-redundancy removal. The N50 value (2784) suggested significant contributions of longer sequences to the total length, indicating data quality and a genome rich in notable genes or specific expression patterns. The Animal Transcription Factor Database (animalTFDB, version 3.0) identified three prevalent transcription factor (TF) types: C2H2 zinc-finger proteins, bHLH helix-loop-helix factors, and other TFs (Fig. 2G). DESeq2 (version: 1.26.0) revealed 285 differentially expressed genes (227 upregulated, 58 downregulated) (Fig. 2H). Cluster analysis highlighted significant differences in expression between the control and MOF-0.5 groups, as depicted in the heatmap. Enrichment analysis via clusterProfiler (version 3.14.3) identified notable Gene Ontology (GO) terms, such as “cell surface” and “extracellular matrix,” emphasizing their relevance (Fig. 2K).

Sequencing analysis revealed a decrease in the expression of the iron metabolism gene TfR2 and an increase in the expression of the osteogenic-related gene BMP2 and its downstream molecules, Runt-Related Transcription Factor 2 (RUNX2), Alkaline Phosphatase (ALP), Collagen Type I Alpha 1 Chain (COL1A1), and Osteocalcin (OCN) (Figs. 2I and 4F), preliminarily validating the hypothesis that MOF-Fe particles promote osteogenesis by inhibiting TfR2 expression and subsequently activating BMP.

### Osteogenic potential of BMSCs Induced by MOF-Fe nanoparticles of varying concentrations

**Fig. 3:**
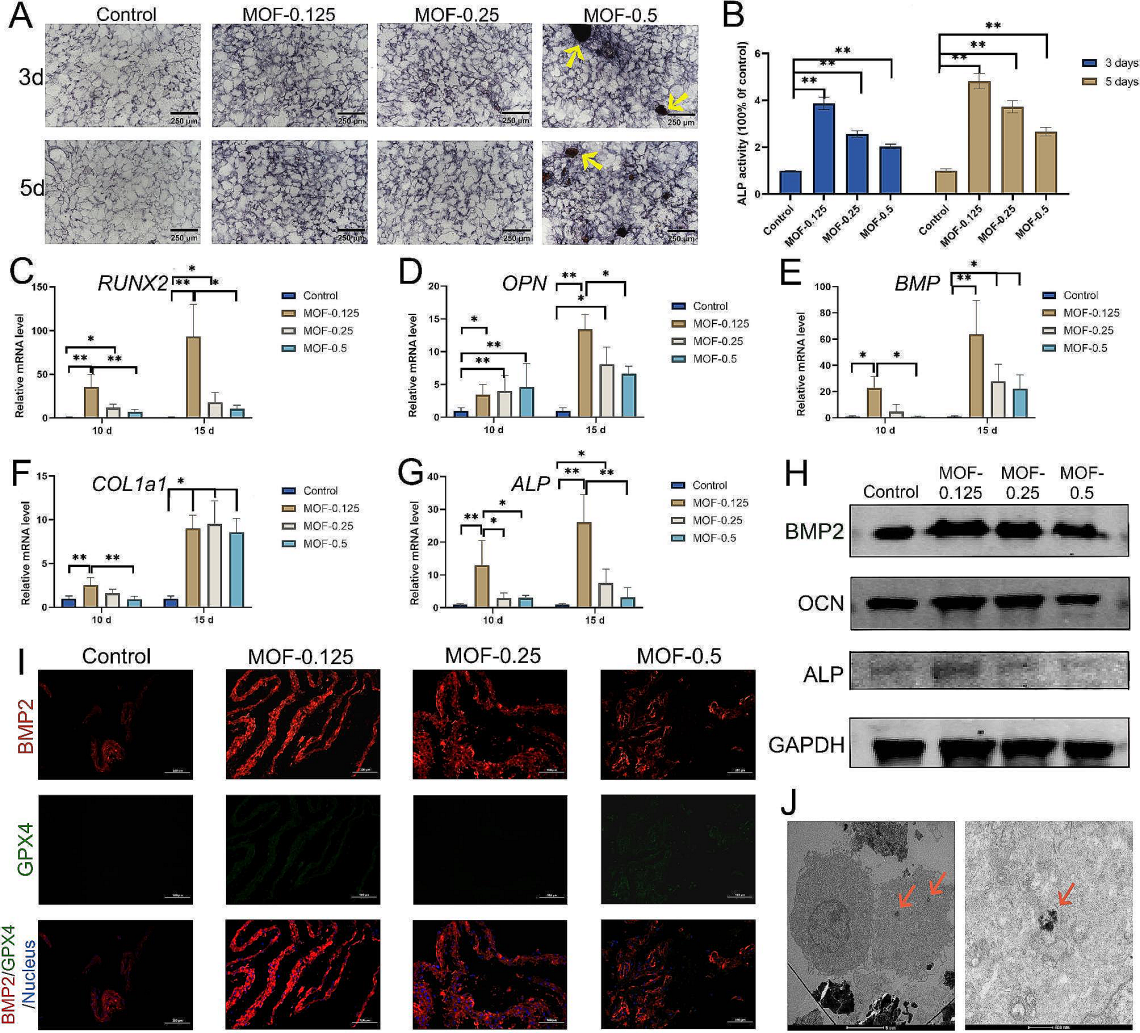
The effect of MOF-Fe nanoparticles on the osteogenic differentiation of bone marrow mesenchymal stem cells. (**A**) ALP staining of bone marrow mesenchymal stem cells. The yellow arrows in the figure indicate the MOF aggregate particles adhered to the cells. (**B**) Quantitative ALP analysis showed that the MOF-0.125 group had the highest ALP activity among the groups with different concentrations of MOF-Fe nanoparticles. (**C**) Expression of the RUNX2 gene. (**D**) Expression of the osteopontin (OPN) gene. (**E**) Analysis of BMP gene expression. (**F**) Expression of the COL1A1 gene. (**G**) ALP gene expression data. (**H**) Changes in BMP2, osteocalcin (OCN), and ALP protein expression. (**I**) Changes in BMP2 and glutathione peroxidase 4 (GPX4) immunofluorescence. (**J**) TEM analysis of the internalization of MOF particles by cells. The data are presented as the means ± SDs (*n* = 3). *p < 0.05, **p < 0.01

This study employed qualitative and quantitative methods to systematically investigate the effects of diverse concentrations of MOF-Fe nanoparticles on ALP activity. After coincubation periods of 3 and 5 days, qualitative analyses of ALP revealed deeper staining within cells in the MOF-0.125, MOF-0.25, and MOF-0.5 groups than in the MOF-free control group, suggesting a pronounced increase in ALP activity (Fig. 3A). A notable increase in ALP enzymatic activity surrounding the MOF-Fe particles was observed. Subsequent quantitative ALP analyses further revealed that MOF-0.125 significantly augmented BMSC ALP activity with increasing incubation time (Fig. 3B).

Subsequently, Alizarin Red staining was performed to determine the potential impact of these nanoparticles on the mineralization of BMSCs. Notably the MOF-0.5 group exhibited the most pronounced mineralization under the influence of MOF-Fe nanoparticles, with a strong accumulation of Alizarin Red staining. Concurrently, the MOF-0.25 and MOF-0.125 groups also displayed superior mineralization compared to that of the control group, with more concentrated mineral nodules observed surrounding the MOF material (Supporting Information Fig. 1). Alizarin Red results showed that MOF-Fe has osteogenic potential, but the optimal osteogenic concentration was contrary to the ALP detection results. Further validation was conducted using qPCR and WB, among other methods.

To further elucidate the impact of MOF-Fe nanoparticles on BMSC gene expression, we performed real-time quantitative polymerase chain reaction (qPCR) to assess the expression levels of the pivotal osteogenic differentiation genes ALP, bone morphogenetic protein (BMP), RUNX2, osteopontin (OPN), and COL1A1, employing glyceraldehyde 3-phosphate dehydrogenase (GAPDH) as the internal reference gene (Fig. 3C-G). Notably, data from Day 10 revealed significant differences in the expression of the ALP, BMP, OPN, and RUNX2 genes between the MOF-0.125 group and the control group. By Day 15, these differences further solidified. Moreover, the COL1A1 gene expression in all three MOF-Fe-treated groups markedly exceeded that in the control group on Day 15, underscoring the potential role of MOF-Fe nanoparticles in osteogenic gene expression.

This study employed Western blot (WB) analysis to assess the impact of MOF-Fe particles on the expression of critical osteogenic proteins in BMSCs (Fig. 3H). The target proteins analysed by WBs included BMP2, OCN, and ALP, and GAPDH was used as an internal reference. The experimental results revealed that the MOF-0.125 group exhibited the highest BMP2, OCN, and ALP protein expression levels, which were significantly greater than those of the untreated control group and the other MOF concentration groups. This finding indicates that MOF-Fe particles at low concentrations significantly increase the osteogenic activity of BMSCs. Immunofluorescence experiments showed that MOF-Fe particles at different concentrations significantly affected the immunofluorescence distribution of BMP2 in BMSCs (Fig. 3I). In the control group, BMSCs not treated with MOF-Fe exhibited lower BMP2 fluorescence signals, suggesting baseline expression of BMP2. Notably, the group treated with MOF-Fe at a concentration of 0.125 mg/mL showed the most intense BMP2 fluorescence signals, indicating that this concentration significantly increased BMP2 expression. While an increase in BMP2 expression was also observed in the groups treated with MOF-0.25 and MOF-0.5, the fluorescence intensity decreased compared with that in the MOF-0.125 group.

Immunofluorescence analysis showed the localization and fluorescence intensity of the GPX4 protein in the different groups. The fluorescence signal of GPX4 in the control group was relatively low, indicating decreased protein expression under baseline conditions. In contrast, GPX4 fluorescence did not significantly increase after treatment with MOF-Fe particles, suggesting that exposure to MOF-Fe particles did not significantly affect GPX4 expression.

Transmission electron microscopy (TEM) analysis revealed successful phagocytosis of MOF-Fe particles by rat BMSCs following 10 days of coculture with 0.5 mg/mL MOF-Fe particles (Fig. 3J). TEM images demonstrated MOF-Fe particle internalization and localized aggregation within intracellular vesicles in the MOF-0.5 group. Increased electron density in the cytoplasm surrounding the particles was observed, likely associated with cellular responses during phagocytosis. The cell nuclei remained intact without significant morphological damage, indicating that bone marrow mesenchymal stem cells can effectively engulf MOF particles while maintaining the integrity of the cell structure.

### Changes in intracellular iron metabolism after coincubation of MOF-Fe with BMSCs

**Fig. 4:**
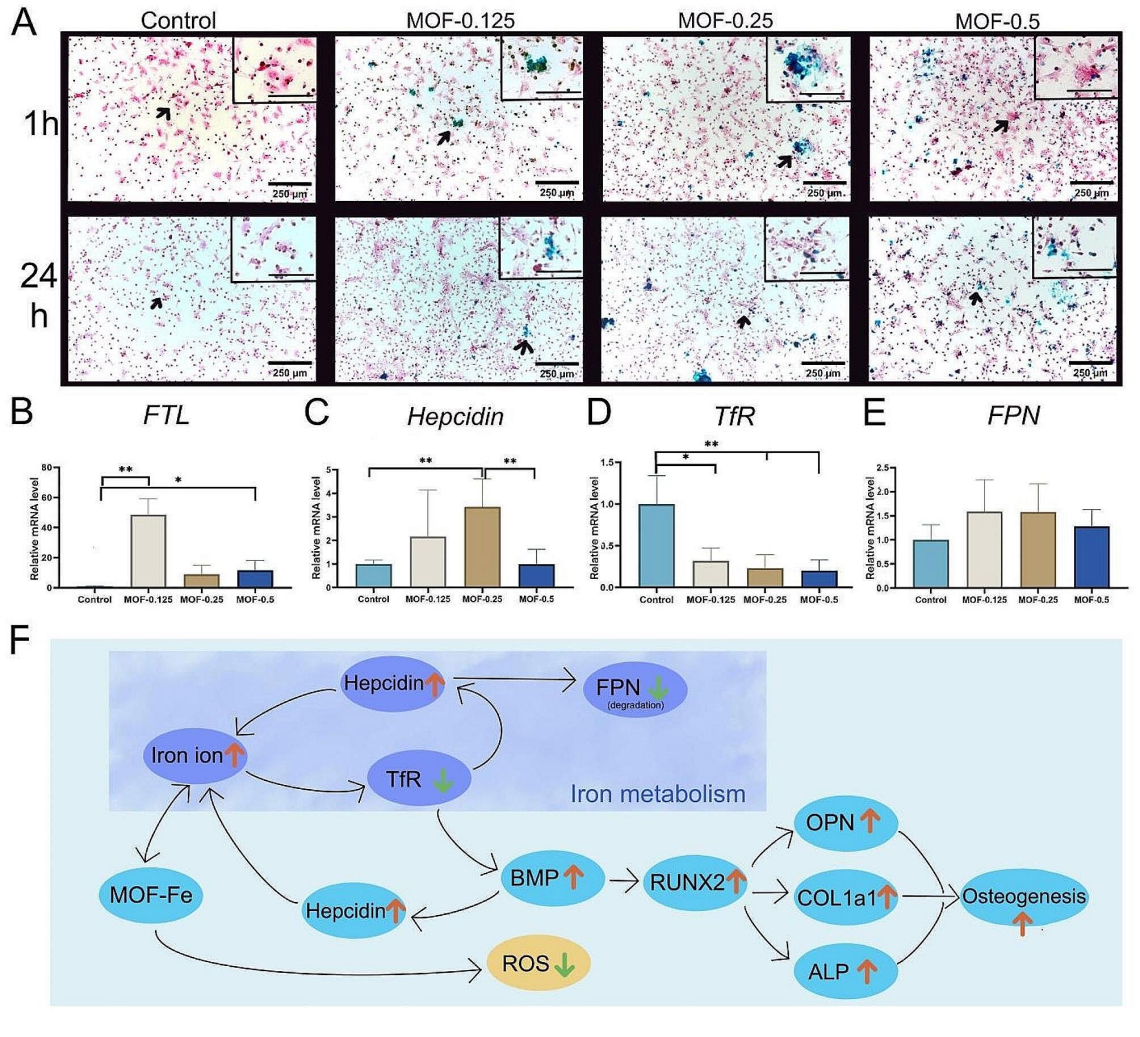
Effects of MOF-Fe Nanoparticles on the Iron Metabolism and Associated Gene Expression of BMSCs. (**A**) Perl’s staining images. A block-like distribution of iron ions is evident after one hour of coincubation, transitioning to a uniform intracellular dispersion at the 24-hour mark. The scale bar in the upper right corner of the magnified image is 125 μm. (**B**) Ferritin light chain (FTL) gene expression. Compared with the control group, the MOF-treated groups exhibited elevated FTL expression. (**C**) Hepcidin gene expression. An increase in hepcidin expression was noted in the MOF-treated groups compared to the control group. (**D**) Transferrin receptor (TfR) gene expression. MOF treatment of BMSCs significantly reduced TfR expression. (**E**) Ferroportin (FPN) expression. (**F**) MOF-Fe particles promote osteogenesis by altering iron metabolism, reducing the expression of TfR2, and activating the BMP signalling pathway. The data are presented as the means ± SDs (*n* = 3). *p < 0.05, **p < 0.01

In the present study, BMSCs were coincubated with various concentrations of MOF-Fe nanoparticles (MOF-0.125, MOF-0.25, and MOF-0.5) for 1 h and 24 h. The intracellular diffusion of iron ions was assessed using Perl’s staining (Fig. 4A). After one hour of staining, iron ions appeared as aggregates across all three experimental groups. However, by 24 h, these ions were uniformly distributed within the cells and no longer formed aggregates. The results indicate that MOF-Fe nanoparticles promote the release of iron ions into cells, achieving a uniform intracellular distribution within 24 h. Importantly, this iron ion release was observed solely in the cells that had come into contact with the MOF-Fe particles.

To further elucidate the effects of iron ions on intracellular gene expression, after 15 days of coincubation, we used qPCR for quantitative analysis of the expression of the ferritin light chain (FTL), TfR, ferroportin (FPN), and hepcidin genes in BMSCs (Fig. 4B-E). The findings revealed that all three MOF-Fe groups showed significantly decreased TfR expression compared to the non-MOF control group. Concurrently, there was an elevation in gene expression for FTL and hepcidin across the MOF groups relative to the control. These observations are consistent with the anticipated iron regulatory alterations, suggesting that cells modulate iron ion metabolism by upregulating hepcidin and FTL expression while downregulating TfR expression in response to iron ion intake (Fig. 4F).

### Assessment of the ROS scavenging capabilities of MOF-Fe at various concentrations

**Fig. 5:**
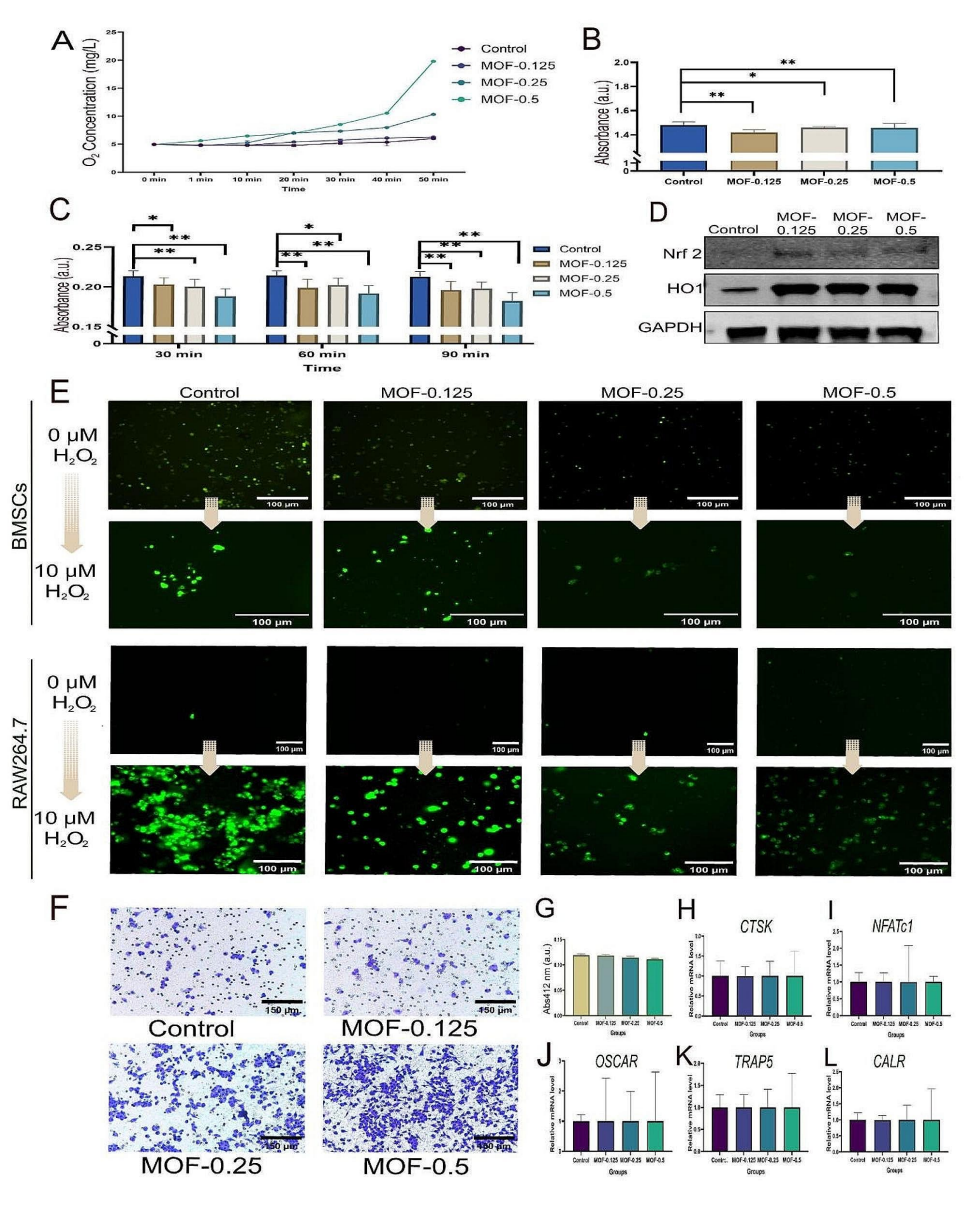
Antioxidant Stress and Reactive Oxygen Species (ROS) Scavenging Effects of MOF-Fe Nanoparticles. (**A**) Oxygen generation from the decomposition of hydrogen peroxide (H_2_O_2_). After exposure to H_2_O_2_, the oxygen generation rate at different MOF-Fe concentrations was evaluated. (**B**) Hydroxyl radical (•OH) scavenging assay. Comparison of •OH radical scavenging efficiency between the MOF treatment and the control groups. (**C**) 1,1-Diphenyl-2-picrylhydrazyl (DPPH) radical scavenging assay. Assessment of the DPPH radical scavenging ability of MOF-Fe nanoparticles at various concentrations. (**D**) Activation of the antioxidant pathway proteins nuclear factor erythroid 2-related factor 2 (Nrf2) and haem oxygenase-1 (HO-1) by different concentrations of MOF particles. (**E**) Fluorescence microscopy images showing ROS levels in BMSCs and RAW264.7 cells, with the fluorescence intensity decreasing as the MOF-Fe nanoparticle concentration increased. (**F**) Transwell assays indicate increased macrophage migration with higher MOF particle concentrations. (**G**) Cocultivation of MOF particles with macrophages had no significant impact on intracellular glutathione levels. **H**-**L**. Cocultivation of MOF particles with macrophages did not significantly affect the expression of mRNAs related to osteoclast differentiation. The data are presented as the means ± SDs (*n* = 3). *p < 0.05, **p < 0.01

In this study, MOF-Fe nanoparticles at different concentrations (MOF-0.125, MOF-0.25, MOF-0.5, and control) were co-incubated with a 3% H_2_O_2_ solution (pH 6.00–7.00), and the pH values of all groups were within the range of 6.00–7.00 (Supporting Information Fig. 2). A concentration-dependent effect was identified by monitoring oxygen evolution within the coincubation system. Compared to that of the control group, a significant acceleration in oxygen generation was observed in the MOF-0.25 group. The MOF-0.5 group showed increased oxygen production, peaking at 19.80 ± 0.10 mg/L at 50 min (Fig. 5A). These findings underscore the efficacy of MOF-Fe nanoparticles in promoting oxygen evolution upon coincubation with hydrogen peroxide, an effect intricately tied to the nanoparticle concentration. Additionally, this study evaluated the efficiency of MOF-Fe nanoparticles in H_2_O_2_ scavenging. Specifically, an 8.94% improvement in H_2_O_2_ scavenging was observed for the MOF-0.125 group at 60 min, which increased to 27.04% for the MOF-0.25 group and 52.11% for the MOF-0.5 group. These data demonstrate a positive correlation between the MOF-Fe nanoparticle concentration and H_2_O_2_ scavenging efficiency.

For •OH, an in-depth exploration of the scavenging efficacy of MOF-Fe nanoparticles towards •OH was conducted. The experimental design included four groups: MOF-0.125, MOF-0.25, MOF-0.5, and a MOF-absent control. Practical outcomes indicated that all MOF-Fe nanoparticle-inclusive groups demonstrated some degree of •OH scavenging. Nonetheless, no pronounced concentration dependence in the scavenging capability of MOF-Fe nanoparticles for •OH was evident (Fig. 5B).

After determining the capacity of MOF-Fe nanoparticles to quench 1,1-Diphenyl-2-picrylhydrazyl (DPPH, pH 6.00–7.00), we found that all MOF-Fe inclusion groups showed decreased DPPH radical levels. Notably, compared with its counterparts, the MOF-0.5 concentration resulted in superior scavenging ability. These experiments corroborate the ability of MOF-Fe nanoparticles to scavenge DPPH radicals, with the effectiveness increasing with increasing MOF-Fe concentration (Fig. 5C).

This study explored the potential of MOF-Fe nanoparticles to quench intracellular ROS fluorescence. The efficacy of MOF-Fe particles at various concentrations was assessed. In the control group, ROS fluorescence was observed using fluorescence microscopy. In contrast, the cells treated with MOF-Fe particles in the experimental group exhibited lower fluorescence intensity, indicating a reduction in intracellular ROS fluorescence (Fig. 5E). BMSCs were treated with 10 µM hydrogen peroxide to induce ROS production, and the ROS fluorescence intensity after treatment with different MOF-Fe particles was analysed. The results demonstrate that the increase in the concentration of MOF-Fe particles is positively correlated with their ability to scavenge ROS. Under hydrogen peroxide-stimulated conditions, control cells without MOF-Fe treatment displayed increased ROS fluorescence signals, suggesting a significant increase in intracellular ROS levels. However, as the concentration of MOF-Fe increased, the ROS fluorescence signals progressively diminished, further validating the effective ROS scavenging action of MOF-Fe particles at higher concentrations.

WB analysis of antioxidant proteins revealed that MOF-Fe particles at varying concentrations significantly impacted the expression of antioxidant stress proteins in BMSCs, particularly affecting the expression of haem oxygenase-1 (HO-1). WB analysis revealed that treatment with MOF-Fe notably increased the protein expression levels of HO-1 and nuclear factor erythroid 2-related factor 2 (Nrf2) (Fig. 5D).

### Impact of different concentrations of MOF-Fe particles on macrophages

#### Transwell assay to investigate changes in RAW 264.7 cell migration behaviour

In the experiment, RAW 264.7 cells were divided into four groups: an untreated control group and three groups treated with MOF-Fe at varying concentrations (MOF-0.125, MOF-0.25, and MOF-0.5) (Fig. 5F). The cell migration of the MOF-0.125 treatment group was similar to that of the control group. However, when the MOF-Fe concentration was increased to 0.5 mg/mL, an increase in cell migration was noted, indicating that MOF-Fe at this concentration effectively promoted the migration of RAW 264.7 cells. These results suggest that MOF-Fe particles at higher concentrations can increase the migration of RAW 264.7 cells.

#### Impact of various concentrations of MOF-Fe particles on ROS changes in RAW 264.7 cells

Transwell assay results indicated that the ability of MOF-Fe particles to attract macrophages increased with increasing particle concentration. This study explored the mechanism driving the migration of RAW 264.7 cells and hypothesized that macrophages might phagocytose some MOF-Fe particles, leading to the disconnection of the organic framework and iron within the MOF-Fe structure, consequently releasing free iron ions and generating ROS. Further investigation was conducted to determine ROS levels in RAW 264.7 cells to validate this theory (Fig. 5E). ROS fluorescence was measured in RAW 264.7 cells coincubated with different concentrations of MOF-Fe particles for five days. In the control group, RAW 264.7 cells exhibited baseline ROS levels as a reference for subsequent comparisons. No significant ROS fluorescence was observed in the MOF-Fe-treated groups. The ROS fluorescence intensity decreased with increasing MOF-Fe concentration after additional hydrogen peroxide stimulation. Overall, the experimental results suggest that the phagocytosis of MOF-Fe particles by macrophages does not lead to ROS production.

#### Impact of various concentrations of MOF-Fe particles on glutathione changes in RAW 264.7 cells

To further investigate whether macrophage phagocytosis of particles triggers oxidative stress responses, we measured glutathione levels in RAW 264.7 cells (Fig. 5G). As glutathione is the principal intracellular antioxidant molecule, its stability is a crucial indicator of the balance of cellular redox states. Monitoring these changes enables the assessment of oxidative stress responses in macrophages after phagocytosis of particles. The experimental results revealed no significant differences in glutathione (GSH) levels in the RAW 264.7 macrophages treated with varying concentrations of MOF-Fe particles. This finding further suggested that MOF-Fe does not cause an increase in ROS levels in RAW 264.7 cells.

#### Impact of various concentrations of MOF-Fe Particles on the Differentiation of RAW 264.7 Cells towards Osteoclasts

qPCR analysis revealed no significant differences in the expression levels of osteoclast-specific genes, such as calreticulin (Calr), cathepsin K (ctsk), nuclear factor of activated T cells, cytoplasmic 1 (NFATc1), osteoclast associated receptor (Oscar), and tartrate-resistant acid phosphatase 5 (TRAP5), in the control group or in the groups treated with various concentrations of MOF-Fe (Fig. 5H-L). This result suggested that within the tested range of MOF-Fe concentrations, MOF-Fe particles did not significantly affect the osteoclast differentiation of RAW 264.7 cells.

### In vivo bone regeneration experiments

**Fig. 6:**
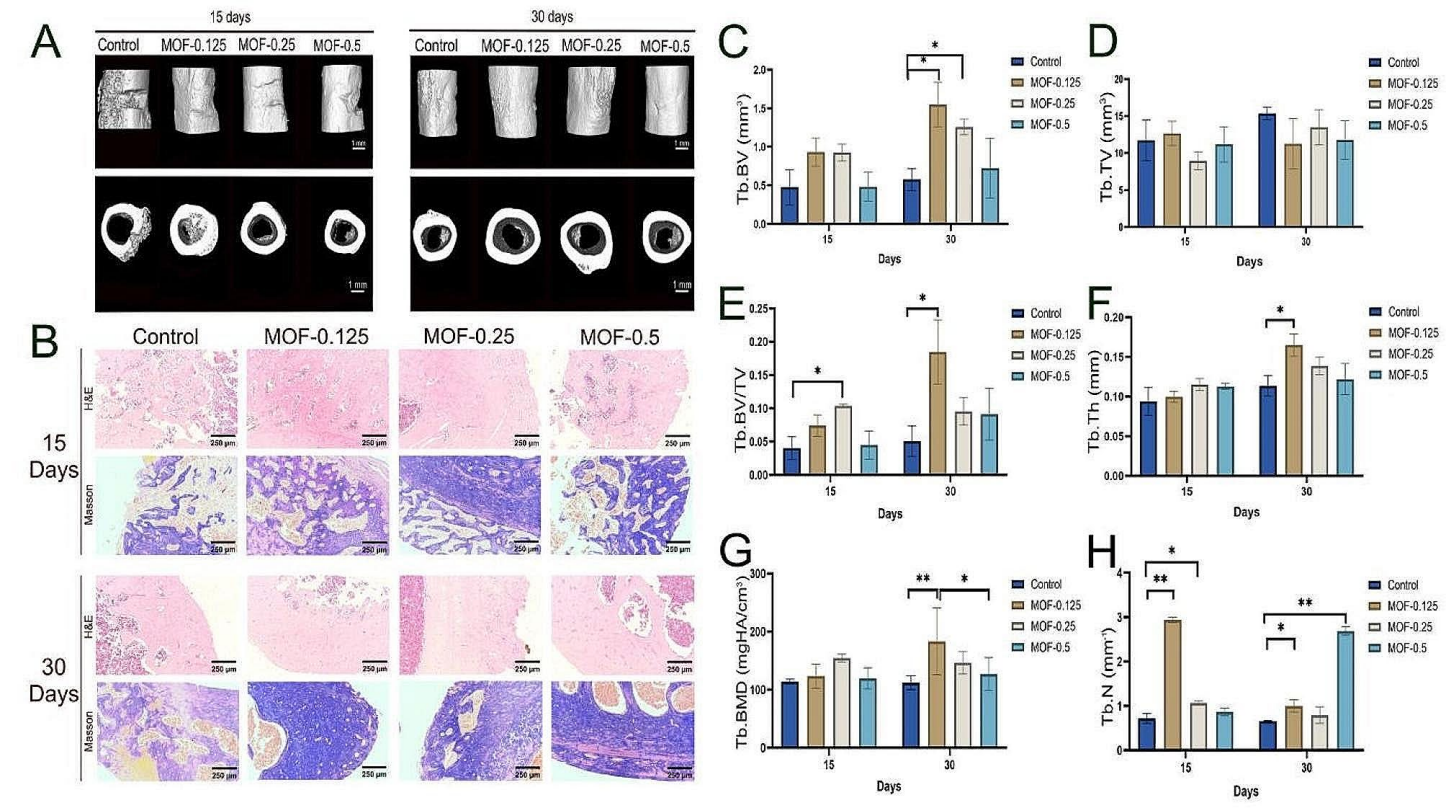
Therapeutic Efficacy of MOF-Fe Nanoparticles in Rat Femoral Bone Defect Restoration. (**A**) Micro-CT imaging: Three-dimensional reconstructions vividly demonstrate the facilitative impact of MOF-Fe nanoparticles on bone tissue regeneration. (**B**) Haematoxylin-eosin (H&E) and Masson’s trichrome staining showing neo-bone tissue morphology and bone collagen maturation status. Notably, in the MOF-0.125 and MOF-0.25 groups, the neo-bone structure exhibited a pronounced thickening compared to that of the control. (**C**) Trabecular bone volume (Tb.BV). (**D**) Trabecular tissue volume (Tb.TV). (**E**) Trabecular bone volume-to-tissue volume ratio (Tb.BV/TV). (**F**) Trabecular thickness (Tb.Th). (**G**) Trabecular bone mineral density (Tb.BMD). (**H**) Trabecular number (Tb.N). The data are presented as the means ± SDs (*n* = 3). *p < 0.05, **p < 0.01

This study employed micro-CT technology to investigate the potential facilitative role of MOF-Fe nanoparticles in restoring rat femoral bone defects. Three-dimensional reconstructions from micro-CT images provided visual insights, revealing the pronounced efficacy of MOF-Fe nanoparticles in promoting bone tissue regeneration and reconstruction. Specifically, with increasing MOF-Fe concentrations, the neo-bone structural morphology progressively approximated completeness, the trabecular arrangement became increasingly ordered, and the bone quality closely resembled that of normal bone tissue (Fig. 6A).

Micro-CT analysis (Fig. 6C-H) revealed that on Day 30, the trabecular bone volume (Tb.BV) significantly increased in the group treated with 0.125 mg/mL MOF-Fe, suggesting that as time progressed, 0.125 mg/mL MOF-Fe may have a more obvious promoting effect on osteogenic activity. In terms of the ratio of bone volume to tissue volume (Tb.BV/TV), 0.125 mg/mL MOF-Fe resulted in a significant increase on Days 15 and 30. In addition, the bone thickness (Tb.Th) increased to a certain extent throughout the experimental period due to the influence of the MOF-Fe concentration. In contrast, regarding the changes in bone mineral density (Tb.BMD), there was no significant change in the MOF-Fe-treated group on Day 15. Nevertheless, by Day 30, the 0.125 and 0.25 mg/mL MOF-Fe treated groups showed an increase, which may indicate that MOF-Fe at these concentrations contributes to the long-term mineralization process. Overall, these results suggest that MOF-Fe particles positively impact bone reconstruction and that this effect is jointly regulated by concentration and time.

In the haematoxylin and eosin (H&E)-stained sections of the neo-bone tissue in the MOF-0.125 and MOF-0.25 groups, there was a notable increase in the amount of neo-bone tissue with a more refined trabecular structure. Masson’s trichrome-stained sections also revealed the bone collagen distribution and maturation state. In summary, both H&E and Masson’s trichrome staining underscore the ability of MOF-Fe nanoparticles to significantly accelerate the repair of rat femoral bone defects, with the MOF-0.125 group exhibiting the most pronounced effects (Fig. 6B).

**Fig. 7:**
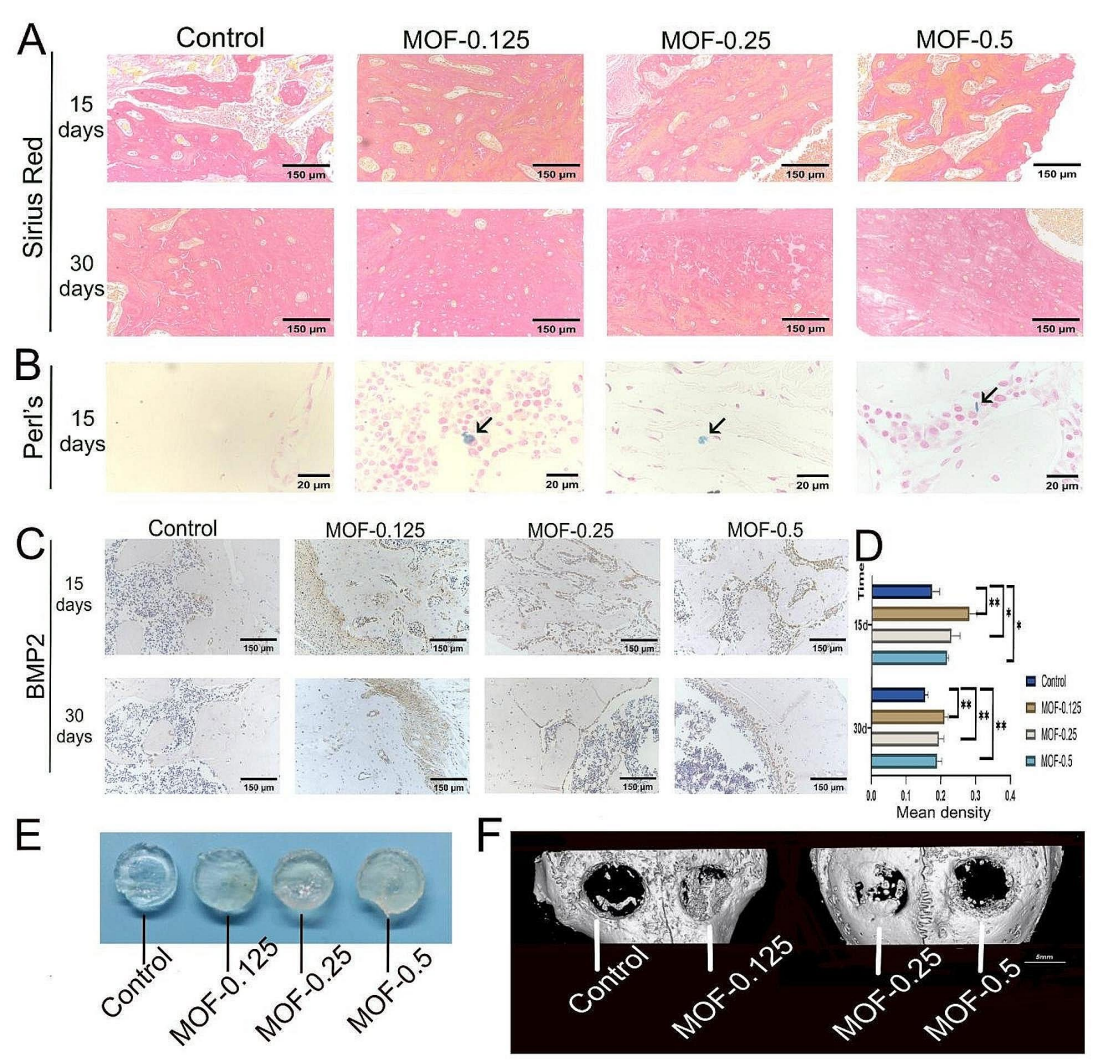
Osteogenic Efficacy of MOF-Fe In Vivo. **A**: Sirius Red staining of femur sections at various time points for the control and MOF-Fe-treated groups at different concentrations. **B**: Femur sections from the control and MOF-Fe-treated groups observed with Perl’s staining, with iron ion deposition indicated by black arrows. **C**: Immunohistochemical staining for BMP2 in femur sections from the control group and the groups treated with various concentrations of MOF-Fe. **D**: Quantitative analysis of BMP2 immunohistochemical staining intensity. **E**: Hydrogel scaffolds containing MOF-Fe at various concentrations. **F**: The efficacy of bone defect repair in rabbit cranial bones 30 days post-implantation with hydrogel scaffolds containing different concentrations of MOF-Fe. The data are presented as the means ± SDs for *n* = 3. *p < 0.05, **p < 0.01

Sirius Red staining highlighted collagen fibre differences in femur sections (Fig. 7A). The control group exhibited a baseline fibre structure and density, while the MOF-0.125 group showed increased density and structural organization. MOF-Fe particles at 0.125 mg/ml demonstrated optimal efficacy, promoting collagen synthesis and arrangement. Perl’s staining showed iron deposition (Fig. 7B), with iron displaying intense blue staining. At 15 days, some sections showed blue staining, while at 30 days, no blue-stained particles were observed. Immunohistochemical analysis of BMP2 expression in femur sections revealed a significant increase in the MOF-0.125 group, indicating effective increases in protein expression. The results showed that 0.125 mg/ml MOF-Fe positively affected the expression of BMP2 in femur sections (Fig. 7C-D).

### Comparison of the repair effects of hydrogel scaffolds containing different concentrations of mof-fe on circular cranial defects in rabbits

In the experiment, New Zealand White rabbits implanted with hydrogel Gel-60 scaffolds mixed with different concentrations of MOF-Fe particles were allowed to recover for 30 days under standard conditions. Following this period, cranial sections with the implanted scaffolds were extracted for further computed tomography (CT) analysis. The results demonstrated that in the control group, where the Gel-60 hydrogel without MOF-Fe particles was used, bone repair was relatively slower, with CT imaging revealing gradual filling of the bone defect area. In contrast, the MOF-0.125-treated group exhibited more pronounced bone regeneration and greater bone density in the CT images, indicating that MOF-Fe particles at this concentration significantly promoted bone repair. Bone repair was faster in the MOF-0.25- and MOF-0.5-treated groups than in the control group (Fig. 7E-F).

## Discussion

Metal-organic frameworks (MOFs) have demonstrated superior performance across various applications, notably in catalysis and sensing [13, 16]. These frameworks offer the advantages of low fabrication costs, remarkable stability, and chemical reactivity [17]. Against this backdrop, this study aimed to further explore the potential applications of MOF-Fe in the biomedical field, focusing on its efficacy in promoting bone defect repair.

Through Perl’s staining studies of MOF-Fe particles, we found that within an hour, the MOF-Fe particles exhibited chunky blue staining at the cell periphery. With time, these blue regions gradually dispersed evenly within the cells. This shift in the staining pattern provides compelling evidence for the release of iron ions from MOF-Fe particles and their successful cellular uptake. Notably, this blue staining appeared only in cells in contact with MOF particles, further suggesting that MOF-Fe primarily exerts its effects through direct cell contact. The qPCR analysis revealed that there was a significant alteration in cellular iron metabolism upon contact with MOF-Fe. Specifically, hepcidin expression increased while TfR expression decreased, accompanied by an increase in FTL expression. Some researchers have posited that elevated iron levels stimulate the expression of BMP-2 and BMP-6 in hepatocytes, suggesting an intersection between iron regulation and BMP pathways [18, 19]. Further research has shown that in the iron regulatory pathway, a reduction in the TfR2 can further activate the BMP pathway [4, 20]. Time series qPCR analysis showed that with increasing MOF particle concentration, the expression of BMP was proportionally amplified. BMP pathway activation stimulates the transcription of the RUNX2 gene, ultimately leading to the upregulation of the OPN, COL1A1, and ALP genes [21, 22], thereby promoting the osteogenic process. A potential mechanism has emerged: MOF particles enhance bone formation by inhibiting TfR2 and activating the BMP pathway. However, the iron metabolism-related gene TfR2 did not significantly differ at different concentrations, but the activation of BMP was greatest at 0.125 mg/mL, possibly because an increase in the MOF concentration can cause a decrease in the pH (Fig. 1E, Supporting Information Fig. 2), and at a concentration of 0.5 mg/mL, although bone formation can still be promoted, the effect is somewhat reduced.

In this study, the principle of the ALP qualitative staining experiment is that the transparent working solution is catalysed by intracellular alkaline phosphatase to produce a deep blue substance. After adding MOF-Fe particles, ALP staining showed a deepening of colour, especially in the MOF-0.125 and MOF-0.5 groups. However, the quantitative analysis of ALP showed that the enzyme activity was highest in the MOF-0.125 group. Both qualitative and quantitative results indicate that MOF can increase ALP activity to promote early osteogenesis. However, the reason for the darker colour in the qualitative staining of the MOF-0.5 group is that there are more opaque MOF particles in the MOF-0.5 group, which have a particular impact on light exposure, resulting in a darker colour than expected. Overall, the results should be based on the quantitative results, considering the ALP activity highest in the MOF-0.125 group.

Further experiments, including qPCR, Western blot, and immunofluorescence detection, all indicate that the MOF-0.125 group most effectively promotes osteogenesis. In contrast, Alizarin Red staining results show that the 0.5 mg/mL group has the most mineralised nodules. Considering the support of multiple experiments for the effect of MOF-0.125, we believe that the MOF-0.125 group has the best effect. The black dots in the Alizarin Red staining results represent aggregated MOF-Fe particles, which are closely bound to the cells, and the particles aggregate on the cell surface. The principle of Alizarin Red staining is that the sodium salt of Alizarin Sulfonic Acid forms an orange-red complex with calcium ions. MOF-Fe may bind to Alizarin Sulfonic Acid, resulting in staining results that differ from other experiments.

In recent years, MOF-Fe has been widely studied for its antitumour properties [23], as MOF-Fe catalyses the decomposition of hydrogen peroxide to produce free hydroxyl radicals to kill tumours. However, in this study, we found that MOF-Fe catalyses the decomposition of hydrogen peroxide to produce oxygen rather than free hydroxyl radicals for the following possible reasons. The role of divalent iron ions in the Fenton reaction is well known. In this reaction, under acidic conditions, Fe^2+^ catalyses the decomposition of H_2_O_2_ to generate hydroxyl radicals (·OH) with strong oxidative ability, which triggers the production of more ROS [5]. In tumours, Fe^2+^ can specifically initiate Fenton or Fenton-like reactions to kill tumours. These strategies applied to MOF-Fe result in the creation of pH-responsive self-catalytic nanosystems [5] based on the dynamic changes in the reaction chemistry of catalytic nanomaterials triggered by the acidic tumour microenvironment. In this study, oxygen was immediately produced upon the addition of MOF-Fe under physiological pH conditions, suggesting that MOF-Fe can decompose hydrogen peroxide to deliver oxygen at physiological pH through ROS scavenging (Figs. 1E and 5E, Supporting Information Fig. 2).

As a type of iron-containing particle, MOF-Fe shares some properties with Fe_3_O_4_ and Fe_2_O_3_ particles. In 2011, Shanhu Liu et al. [24] reported the peroxidase-like activity of Fe_3_O_4_, which can act as a catalyst to decompose hydrogen peroxide. In 2012, Ning Gu et al. [25] verified the pH-dependent dual-enzyme activity of iron oxide particles in an intracellular environment. Specifically, under neutral pH conditions (e.g., cytoplasmic conditions), iron oxide particles can catalyse the conversion of hydrogen peroxide into oxygen and water, exhibiting peroxidase-like activity. In contrast, under acidic conditions (e.g., lysosomal conditions), they can decompose hydrogen peroxide into highly toxic free hydroxyl radicals. Therefore, it is speculated that the catalytic principle of MOF-Fe is similar, but it still needs to be verified according to specific conditions. Upon further investigation into the ROS scavenging ability of MOF-Fe, we found that the experimental group treated with MOF-Fe showed effective elimination of hydrogen peroxide from ROS. Moreover, the experimental data indicated that as the concentration of MOF particles increased, the concentration of free radicals decreased. The DPPH free radical scavenging experiment supported this assertion, revealing an increased neutralizing ability of higher MOF particle concentrations towards DPPH free radicals. The amalgamation of experimental data underscores the potential of MOF particles for ROS scavenging, especially for promoting the decomposition of hydrogen peroxide. Intracellular ROS fluorescence analysis further supported this conclusion, clearly highlighting the immense application value of MOF particles in ROS scavenging.

In subsequent animal experiments, osteological analysis of the experimental group rats revealed more enlightening details about MOF-Fe. Compared to those of the control group, the femoral cortex of the exploratory group rats showed a significant increase in bone density. Moreover, increased bone thickness was observed under low MOF concentrations through H&E and Masson’s trichrome staining. Micro-CT analysis further confirmed these observations. We concluded that MOF-Fe nanoparticles significantly promote new bone formation. In this study, Perl’s staining was performed on animal tissue sections to detect the residual MOF-Fe. The results showed that blue-stained particles were observed in some sections only at 15 days, while no blue-stained particles were found in the sections at 30 days, suggesting that MOF-Fe may undergo natural degradation. In our iron release experiment, the results verified that MOF-Fe releases iron slowly (Fig. 1J), reducing the likelihood of iron overload in the organism.

A limitation of this study is that at concentrations of 0.25 and 0.5 mg/ml, MOF-Fe particles significantly increased the migration of RAW 264.7 macrophages. However, this study did not directly investigate the effect of MOF-Fe particles on the polarization state of macrophages, particularly whether these particles can affect the polarization state of macrophages and whether this effect is related to their application in bone tissue engineering. In future research, we will focus on further exploring the effects of MOF-Fe particles on the function of macrophages and elucidate their role in regulating osteogenesis while modulating inflammation levels.

## Conclusions

This study investigated the osteogenic effects of MOF particles and their ability to scavenge ROS. The experimental data suggest that MOF-Fe particles can activate the BMP pathway by inhibiting TfR2, demonstrating good potential for promoting bone formation. Regarding their ability to scavenge ROS, MOF particles exhibited good efficacy in reducing species such as hydrogen peroxide. The outstanding ability of MOF-Fe to eliminate ROS and increase the activity of osteogenic cells highlights its immense potential as a novel bone-inducing material.

### Electronic supplementary material

Below is the link to the electronic supplementary material.

Supplementary Material 1

Supplementary Material 2

Supplementary Material 3

## Data Availability

The datasets used and/or analysed during the current study are available from the corresponding author on reasonable request.
