# Supplementary material for: Biomimetic peroxidase MOF-Fe promotes bone defect repair by inhibiting TfR2 and activating the BMP2 pathway
Source: Biol Direct. 2024 Apr 23;19:30. doi: 10.1186/s13062-024-00473-2 (PMC11036606; doi:10.1186/s13062-024-00473-2)
Supplement: Supplementary file 1 — Supplementary Material 1 [file 13062_2024_473_MOESM1_ESM.docx]

Supporting Information

**Biomimetic Peroxidase MOF-Fe Promotes Bone Defect Repair by Inhibiting TfR2 and Activating the BMP2 Pathway**

*Yaxin Xue^#^; Wei Xu^#^; Danyang Zhao^#^; Zijing Du; Hao Jiang; Hao Lv; Dong Zhang; Zhencheng Yu; Yi Cao^*^; Dong Han^*^*

**Materials and Methods**

*Preparation and Characterisation of MOF-Fe*

The synthesis of iron-based metal-organic frameworks (MOF-Fe) was achieved through solvothermal techniques. Initially, 2.7676 mmol of 2-amino terephthalic acid (BHC-NH2; Macklin, China) was mixed with an equivalent amount of ferric chloride hexahydrate powder (FeCL_3_·6H_2_O; Macklin) in 60 mL of N, N-dimethylformamide (DMF; Macklin). At 26°C, this mixture was stirred at 1000 rpm for 1 hour using a magnetic stirrer (Joanlab, China). Subsequently, the mixture was transferred to a hydrothermal kettle with a capacity of 100 mL and heat-treated at 100℃ for 24 hours. Upon completion and complete cooling, the product was extracted from the reactor and washed three times with DMF at 3000 rpm for 10 minutes at 26°C, followed by three analogous washes with anhydrous ethanol.

Scanning electron microscopy (SEM; ZEISS, Germany) observed the sample's microstructure. To thoroughly investigate the stability of the MOF-Fe material, 50 mg of MOF-Fe was mixed with 30 mL of simulated body fluid (SBF; Pythonbio, China) and then shaken on a shaker bed at 500 rpm for 30 days. After centrifugation, it was again observed with SEM. In addition, the particle size distribution of MOF-Fe nanoparticles was determined using a laser particle size analyser (Beckman, USA). To evaluate pH variations, concentrations of SBF suspension mixed with MOF-Fe nanoparticles at different densities were set at 0.125, 0.25, 0.5, and 0 mg/mL, correlating respectively to MOF-0.125, MOF-0.25, MOF-0.5 groups, and a control group. On days 0, 1, 4, 6, and 8 of the experiment, pH measurements were taken with a pH meter (Mettler Toledo, USA) to monitor the acidity or alkalinity of the suspension.

Furthermore, to measure the zeta potential of samples from each group, MOF-Fe nanoparticles at different concentrations were initially dispersed in deionised water, followed by measurement at a constant temperature of 25°C using a zeta sizer nano ZS (Malvern Instruments, UK). An extensive characterisation of MOF-Fe also involved mapping images of four elements: carbon (C), oxygen (O), nitrogen (N), and iron (Fe). This step aimed to visually depict these elements' distribution within MOF-Fe nanoparticles.

Fourier transform infrared (FT-IR) spectroscopy was utilised to identify the composition of chemical bonds. This test was followed by the elemental composition of the samples using energy-dispersive X-ray spectroscopy (EDS). Moreover, X-ray Photoelectron Spectroscopy (XPS) afforded deeper insight into the samples' surface elemental composition and chemical states. Nitrogen adsorption-desorption experiments were performed under liquid nitrogen at 77 K and vacuum conditions using an automated surface area analyser (Micromeritics, USA) to probe the porous properties of the material. Based on this, the material's total surface area and pore diameter were further calculated. This study evaluates the release performance of MOF-Fe nanoparticles for free iron ions under various conditions. The different concentrations of MOF-Fe nanoparticles (0.125 mg/ml, 0.25 mg/ml, 0.5 mg/ml) were co-incubated with dulbecco's modified eagle medium (DMEM; HyClone, USA). The release of iron ions was subsequently monitored over varying durations (1, 4, 7, 10, 20, and 30 days). Specific experimental procedures entailed mixing MOF-Fe nanoparticles with a complete culture medium at designated concentrations and incubating for set durations. At each predetermined sampling interval, samples, post-centrifugation at 3000 rpm for 10 minutes, were processed by collecting the supernatant. The samples subsequently underwent acid dissolution, with the final concentrations of free iron ions determined using inductively coupled plasma optical emission spectroscopy (ICP-OES).

*Cultivation and Identification of BMSCs*

Two 4-week-old male SD rats were selected to acquire BMSCs. Following euthanasia, femurs from their hind legs were procured, and bone marrow cavities were flushed using a sterile 1 ml syringe combined with saline solution, thus obtaining a bone marrow tissue suspension. This suspension was subsequently placed in high-glucose DMEM, enriched with 10% fetal bovine serum (FBS; Gibco, USA) and supplemented with 100 U/mL penicillin and 100 µg/mL streptomycin (HyClone), reserved for subsequent experiments.

Tri-lineage differentiation and cellular phenotype identification methods were employed for BMSC cellular identification. In osteogenic differentiation assays, BMSCs were induced in a complete high-glucose medium replete with ten nM dexamethasone (Sigma, USA), ten mM β-glycerophosphate (Merck, USA), and 50 µg/mL vitamin C (Sigma). After 2-3 weeks of cultivation, microscopic observation was facilitated by alizarin red staining (Solarbio, China). On the other hand, adipogenic differentiation employed the rat BMSC adipogenic differentiation kit (Oricell, China); following lipid droplet formation, further staining with red oil (Oricell) was conducted. For chondrogenic differentiation, BMSCs were cultivated in chondrogenic differentiation medium containing 200 ng/mL Transforming Growth Factor Beta (TGF-β, Nanoprotein, USA), 5 µL insulin-transferrin-selenium (ITS, Invitrogen, USA), 4 x10^-8^ g/mL dexamethasone, and 0.05 mg/mL vitamin C. After 2-3 weeks, paraffin sections were prepared and stained with alcian blue (Sigma) for the observation of blue cartilage matrix. Subsequent flow cytometry was utilised to identify surface markers of rat BMSCs, which involved incubating 1 x 10^6^ cells with fluorescent-labelled antibodies against CD44 (Abcam, UK), CD90 (Abcam, UK), and CD45 (Abcam, UK). Herein, CD44 and CD90 are positive markers for mesenchymal stem cells, whereas CD45 aids in excluding potential hematopoietic contamination. Post 20-minute incubation in a darkened environment at 4℃, cells were analysed via flow cytometry. Expected results from this flow cytometric procedure would manifest a significant positive expression rate for CD44 and CD90 and a low positive expression rate for CD45, affirming successful BMSC isolation and cultivation.

*Post-exposure Evaluation of BMSC Viability in Contact with MOF-Fe Particles*

For this study, suspensions were prepared by incorporating MOF-Fe into the complete culture medium, specifically at concentrations of 0.125 mg/mL, 0.25 mg/mL, and 0.5 mg/ml. Concurrently, a control group devoid of MOFs was established, designated as MOF-0.125, MOF-0.25, MOF-0.5, and the control group. After incubating at four °C for 24 h, the supernatant was collected. After that, 100 µL of this supernatant was introduced into each well of a 96-well plate, supplemented with rat BMSCs (1 × 10^4^ cells/mL, 100 µL/well) and incubated further for 1 to 5 days at 37°C in a 5% CO_2_ environment. After incubation, 10 µL CCK-8 (Sigma) was added to each well for 2 hours to assess cell viability, with optical density (OD) subsequently measured at 450 nm. Use pH test strips to detect the pH values of the MOF-0.125 group, MOF-0.25 group, MOF-0.5 group, and control group.

Given the potential impact of MOFs-Fe on BMSC morphology, experiments were designed to delve into this phenomenon. Initially, rat BMSCs (2.5 × 10^5^ cells/mL) were seeded in a 24-well plate for five days and subsequently co-incubated for one day with four varying concentrations of MOF suspensions, including MOF-0.125, MOF-0.25, MOF-0.5, and a control group without MOF. After washing thrice with 1×PBS, cells were fixed with 4% paraformaldehyde for 10 minutes and treated with 0.5% Triton-X100 (Sigma) for 5 minutes. Following the manufacturer's protocol, the trite-phalloidin staining solution (Maokangbio, China) was utilised for cell re-suspension and incubated in the dark for 30 minutes. After a further wash with 1×PBS, cells were stained with 4',6-diamidino-2-phenylindole (DAPI, Biosharp, China) for 30 seconds. After these procedures, morphological alterations of cells post-contact with MOF nanoparticles were investigated utilising a fluorescence microscope (Zeiss, Germany).

*The transcriptomic sequencing and analysis of BMSCs in response to MOF-Fe particles*

This investigation examined the transcriptomic effects of MOF-Fe on BMSCs. The experimental design comprised two cohorts: a control group without MOF exposure and the treatment groups subjected to MOF at 0.5 mg/ml concentrations. Cells were incubated with the respective MOF concentrations for ten days. After incubation, total RNA was extracted utilising a Trizol reagent (Thermo Fisher, USA). Subsequently, RNA-sequencing (RNA-seq) technology was used to acquire transcriptomic data for each group. During the data processing and analysis phase, initial quality control was executed to eliminate low-quality sequences and contaminants, followed by sequence alignment mapping reads to the reference genome. Bioinformatics tools were utilised to conduct differential gene expression analysis to elucidate the potential impact of MOF treatment on the gene expression patterns in BMSCs.

*Assessment of Osteogenic Potential in BMSCs Induced by MOF-Fe Particles of Varying Concentrations*

*ALP Activity Assay*

Qualitative and quantitative Alkaline Phosphatase (ALP) activity was evaluated to discern early osteogenic activities across different groups. BMSCs (1.2 x 10^6^ cells/mL) were cultured in a 6-well plate for five days, after which they were co-cultured with MOF-0.125, MOF-0.25, MOF-0.5, and the control group for three and five days, respectively. For the qualitative assessment, BMSCs were stained using the working solution from the BCIP/NBT alkaline phosphatase colour development kit (Beyotime) and observed under a microscope for colourimetric changes. The subsequent quantitative analysis entailed the preparation of the chromogenic substrate and standard solution following the Alkaline Phosphatase Assay Kit's protocol (Beyotime). Cells were lysed using 0.1% Triton X-100 (Thermo Fisher Scientific, USA), and the supernatant was obtained after centrifugation at 12,000 rpm for 3 minutes. This supernatant was mixed with the chromogenic substrate in a 1:1 ratio (50 µL each) and incubated at 37℃ for 10 minutes in a 96-well plate. Absorbance was measured at 405 nm using a spectrophotometer to determine ALP activity.

*Alizarin Red Staining*

BMSCs (2.5 x 10^5^ cells/mL) were co-cultured with four concentrations of MOF-Fe particles (MOF-0.125, MOF-0.25, MOF-0.5, and Control) in a 24-well plate for 15 and 30 days. At the culmination of the incubation, cells were fixed with 4% paraformaldehyde for 10 minutes and rinsed thrice. Cells were stained with alizarin red solution (Solarbio) for 2 hours, washed with 1× PBS, and inspected under a light microscope.

*Evaluation of Osteogenic Gene Expression*

BMSCs were co-cultured with MOF-0.125, MOF-0.25, MOF-0.5, and the control group, after which the real-time quantitative polymerase chain reaction (qPCR) analysis was carried out. BMSCs were prepared and co-incubated with four different MOF concentrations for 10 and 15 days. Total RNA was extracted using a TRIzol reagent. RNA concentration and purity were ascertained via nanodrop (Thermo Scientific, USA). The reverse transcription process involved synthesising cDNA using 1μg of total RNA and the prime script RT master mix (Takara, Japan). The qPCR analysis was performed on cDNA using SYBR premix ex taq II (Takara) and specific primers. Relative gene expression levels were computed via the 2^−ΔΔCT^ method, considering glyceraldehyde 3-phosphate dehydrogenase (GAPDH) as the housekeeping gene. Genes assessed included bone morphogenetic protein (BMP), runt-related transcription factor-2 (RUNX2), ALP, osteopontin (OPN), and collagen type I alpha one chain (COL1A1) (Supporting Information Table 1).

**Supporting Information Table 1.** Primer sequences of BMSCs used for qPCR analysis (Rat).

| Genes | Forward (5'-3') | Reverse (5'-3') |
| --- | --- | --- |
| ALP | AAGGCTACGACACCGTCACT | TCCTTATCATCCGCCGGTGT |
| BMP | TATGCTCGACCTGTACCGCC | TCTGGAAGTTCCTCGATGGCT |
| RUNX2 | CATGGTGGAGATCATCGCGG | ACCTCTCCGAGGGCTACAAC |
| COL1A1 | CTTCAGCTTCCTGCCTCAGC | GGCTCAGGCTCTTGAGGGTA |
| OPN | CAGCATTTCGCTTCTGTTCTTT | AGTTTGCCTGCCTCTACATAC |
| GAPDH | TTGTGGATCTGACATGCCGC | AGCCCAGGATGCCCTTTAGT |
| TfR | GAGAAAGCCACAAGCCAAAC | GCCACAACTCACTGGACTTA |
| FTL | CTTCGCGGTTAGTTCCATACT | CCAGAGAGAGGTAGGTGTAAGA |
| Hepcidin | GCTTAGAGGGAGACAGCATTT | TTCCTCCTGGGCTCTATTAGT |
| FPN | TGATGGGAGCATCAGCAATAA | GAACAGACCAGTCCGAACAA |

*Influence of MOF-Fe Particles at Varied Concentrations on Osteogenic Protein Expression in BMSCs*

This study employed Western Blot (WB) analysis to assess the impact of MOF-Fe particles on the expression of critical osteogenic proteins in BMSCs. The experimental protocol included four groups: an untreated control and three varying MOF-Fe concentrations of 0.125, 0.25, and 0.5 mg/ml. Each cohort of cells was incubated continuously with the respective MOF-Fe concentrations for ten days. Post-incubation, cells were lysed using RIPA buffer (biosharp) containing protease inhibitors, and proteins were extracted. Protein concentrations were quantified using a BCA Protein Assay Kit (biosharp) to ensure equal protein loading for subsequent WB experiments. Target proteins analysed included BMP2, Osteocalcin (OCN), ALP, and GAPDH as the loading control. Samples were subjected to SDS-PAGE (biosharp) and transferred onto polyvinylidene difluoride (PVDF) membranes. These membranes were then blocked and incubated with antibodies targeting BMP2 (Abcam, 1:1000), OCN CST, 1:1000), ALP (CST, 1:1000), and GAPDH (Abcam, 1:1000). Signal detection was performed using an Enhanced Chemiluminescence (ECL) detection system after binding with the appropriate secondary antibodies (HRP Anti-Rabbit IgG antibody, Abcam, 1:2000).

*Impact of MOF-Fe Particle Concentration on BMP2 and GPX4 Distribution in BMSC Cells*

This investigation aimed to evaluate the effect of MOF-Fe particles on BMP2 expression in BMSCs. The experimental design included a control group devoid of MOF and three groups treated with MOF-Fe at concentrations of 0.125, 0.25, and 0.5 mg/ml. Initially, BMSCs were co-cultured with the respective concentrations of MOF-Fe under optimal cell culture conditions for ten days, during which the cells were maintained in an incubator with constant temperature, humidity, and 5% CO_2_ atmosphere. Upon completion of the incubation period, the adherent BMSCs were detached using trypsin digestion, followed by centrifugation and washing to remove the medium and non-adherent cells. Subsequently, the cells were fixed, dehydrated, and embedded in paraffin blocks. For immunofluorescence staining, sections from the paraffin blocks were deparaffinised and rehydrated, then subjected to antigen retrieval to enhance antibody binding. After that, the sections were incubated with primary antibodies specific to BMP2 (Abcam) and Glutathione Peroxidase 4 (GPX4, CST, US), followed by incubation with fluorescently labelled secondary antibodies. Finally, fluorescence microscopy was employed to observe and capture images, allowing for the assessment and comparison of the expression across the different treatment groups of BMSCs.

*Observation of Rat BMSC Cells phagocytising MOF-Fe Particles*

In the study of rat BMSCs phagocytising MOF-Fe particles, the transmission electron microscopy (TEM, Zeiss) observation method is described as follows: BMSCs were extracted and cultured under aseptic conditions to an appropriate density. Subsequently, MOF-Fe particles were added to the culture medium at a 0.5 mg/mL concentration to establish the MOF-0.5 experimental group. After co-culturing for ten days, cells were washed with PBS to remove unphagocytised MOF particles and fixed and embedded to prepare TEM samples. Then, TEM was utilised to observe and record the morphological changes of MOF-Fe particles phagocytised inside BMSCs, assessing their phagocytic efficiency and intracellular distribution of particles.

*Alterations in Intracellular Iron Metabolism Following Co-incubation of MOF-Fe with BMSCs*

*Perl's Staining to Investigate Iron Dynamics*

Perl's staining method was employed as one of the detection measures to investigate the iron release dynamics of MOF-Fe nanoparticles at different concentrations within BMSCs. Initial experimentation involved the adhesion of BMSCs (1.2 x 10^6^ cells/mL) to a six-well plate, followed by co-incubation with diverse concentrations of MOF-Fe nanoparticles (0 mg/ml, 0.125 mg/ml, 0.25 mg/ml, and 0.5 mg/ml) for time intervals of 1 and 24 hours at established conditions of 37℃ in a 5% CO_2_ atmosphere. After the incubation, cells were rinsed with PBS and fixed using 4% formaldehyde for 30 minutes. Cells were stained for 30 minutes employing Perl's staining kit (Solarbio), during which a reaction between Perl's staining solution and iron ions yielded the formation of potassium hexacyanoferrate, rendering iron-containing particles a blue hue. For optimal microscopic visualisation, further steps entailed washing the cells with PBS to remove excess dye, counterstaining with Eosin stain solution (Solarbio), and observing under a microscope.

*qPCR Analysis for In-depth Insight into Iron Metabolism Post Interaction*

This analysis employed qPCR to analyse changes in iron-related gene expression in rat BMSCs to gain comprehensive insights into the specific impact on iron metabolism post-interaction of MOF-Fe nanoparticles with BMSCs. Different concentrations of MOF-Fe nanoparticles, namely 0 mg/ml, 0.125 mg/ml, 0.25 mg/ml, and 0.5 mg/ml, were introduced and co-cultured with BMSCs (1.2 x 10^6^ cells/mL) in a six-well plate for 15 days at 37℃ in a 5% CO_2_ environment. Following incubation, qPCR reactions were undertaken using primers specific to iron-associated genes such as ferroportin (FPN), transferrin receptor (TfR), ferritin light chain (FTL), and hepcidin. Analysis of the qPCR data, employing the 2^-ΔΔCt^ method, compared the gene expression variance between different MOF-Fe nanoparticle concentration groups and the control group, unveiling the potential impact of MOF-Fe nanoparticles on the iron-related gene expression in BMSCs (Supporting Information Table 1).

*Assessment of Antioxidant Stress Capabilities of MOF-Fe at Various Concentrations*

An array of systematic assays was executed to elucidate the antioxidant capabilities of MOF-Fe nanoparticles at various concentrations.

*H_2_O_2_ Scavenging Efficacy*

An initial 3% H_2_O_2_ solution (Jianning, China) was prepared in batches of 5 mL each to discern the scavenging capacity of MOF-Fe nanoparticles for hydrogen peroxide at diverse concentrations. After this, incremental quantities of MOF nanoparticles were introduced, resulting in the designated groups MOF-0.125, MOF-0.25, and MOF-0.5, in addition to a control group. Use pH test strips to measure the pH values of the MOF-0.125 group, MOF-0.25 group, MOF-0.5 group, and the control group. A portable dissolved oxygen meter (Lianchuang, China) was employed throughout the experimental duration to monitor the oxygen concentration at ten-minute intervals. In conclusion, a comparative analysis of oxygen concentrations across groups facilitated calculating the scavenging efficiency of MOF-Fe nanoparticles for hydrogen peroxide.

*DPPH Radical Scavenging Assay*

To investigate the scavenging capability of MOF-Fe nanoparticles towards DPPH radicals, a 50 μg/mL DPPH• ethanol solution was initially prepared, with 2 mL allocated for each group. Subsequently, MOF-Fe nanoparticles of varying concentrations (0 mg/ml, 0.125 mg/ml, 0.25 mg/ml, 0.5 mg/ml) were added to the respective DPPH• ethanol solutions. All samples were then left to react in the dark. Absorbance at 519 nm was measured every 30 minutes until the 90-minute mark. Differences in absorbance levels were subsequently utilised to infer the DPPH scavenging efficiency at each MOF-Fe concentration.

*•OH Radical Scavenging Assay*

The scavenging potential of MOF-Fe nanoparticles against •OH was evaluated using the Fenton reaction to generate •OH radicals. In this reaction, a 0.1 mmol/mL solution of FeSO_4_ (Merck) was combined with 100 mM H_2_O_2_, where the Fe^2+^ ions and hydrogen peroxide interacted to produce the requisite radicals. MOF-Fe nanoparticle solutions of varying concentrations were then co-incubated with the radical mixture for an hour. After incubation, salicylic acid (100 mg/mL; Merck) was added due to its reactivity with •OH, producing 2,3-dihydroxybenzoic acid, exhibiting characteristic absorbance at 510 nm. This absorbance was subsequently gauged to determine the radical scavenging efficacy quantitatively.

*Intracellular ROS Scavenging Assessment*

Cells were seeded in 6-well plates at 1.2×10^6^ cells/well and cultivated for five days. After this, cell cultures were exposed to nanoparticulate MOF-Fe solutions at concentrations delineated as MOF-0.125, MOF-0.25, and MOF-0.5, and a control group was co-cultivated for 24 hours. Post incubation, in adherence to the reactive oxygen species (ROS) assay kit (Solarbio) guidelines, 2', 7'-dichlorofluorescein diacetate (DCFH-DA) was introduced to the cells, followed by a further incubation of 30 minutes in the dark. Cells were then washed with PBS, ensuring complete removal of unreacted DCFH-DA. Then, this process involved the detection of intracellular ROS fluorescence intensity via confocal microscopy.

To elucidate more clearly, cells from various groups were stimulated with ten µM hydrogen peroxide for one hour, after which ROS fluorescence intensity was reassessed.

*Influence of Varying Concentrations of MOF-Fe Particles on the Expression of Antioxidative Stress Proteins in BMSC Cells*

This study investigated the influence of MOF-Fe on the expression of antioxidative stress proteins in BMSCs using the WB technique. The experimental setup included a control group without MOF treatment and three groups treated with MOF at concentrations of 0.125, 0.25, and 0.5 mg/ml. Each group of BMSCs was co-cultured with corresponding concentrations of MOF particles for ten days. This experiment assessed the expression of antioxidative stress proteins, including Heme Oxygenase-1 (HO-1) and Nuclear Factor Erythroid 2–Related Factor 2 (Nrf2). HO-1, an inducible enzyme, gets activated under oxidative stress conditions, playing a crucial role in maintaining cellular homeostasis and antioxidative defence. Nrf2, a transcription factor, centrally regulates antioxidative stress response by activating the expression of various antioxidative enzymes, including HO-1. Therefore, the expression levels of HO-1 and Nrf2 serve as critical indicators of the cellular antioxidative stress capability. During the experimental process, total protein was extracted from the cells of each group and quantified. Subsequently, the proteins were separated via SDS-PAGE electrophoresis and transferred onto Polyvinylidene Difluoride (PVDF) membranes. Proteins on the membranes were incubated with specific antibodies against HO-1 (CST), Nrf2 (CST), and Glyceraldehyde 3-phosphate Dehydrogenase (GAPDH, Abcam) as the internal control. Appropriate secondary antibodies and chemiluminescence methods were used for protein signal detection. By comparing the expression levels of HO-1 and Nrf2 across different treatment groups, the impact of MOF-Fe on the antioxidative stress capacity of BMSCs was evaluated, thereby revealing its potential role in modulating cellular antioxidative mechanisms.

*Impact of MOF Particles at Diverse Concentrations on Macrophages*

In this study, the RAW 264.7 macrophage cell line, sourced from the Cell Bank, Chinese Academy of Sciences (TCM13), was cultured in high-glucose DMEM supplemented with 10% fetal bovine serum and 1% penicillin-streptomycin to maintain an optimal growth environment. The cells were incubated at 37°C in a 5% CO_2_ atmosphere until they reached the logarithmic growth phase.

*Transwell Assay Investigating Migration Behaviour Alterations in RAW 264.7 Cells*

In the Transwell assay of RAW 264.7 macrophage cells, a 48-well transwell plate (8 um, biosharp) was utilised, establishing four experimental groups: a control group without MOF and three MOF-treated groups at concentrations of 0.125, 0.25, and 0.5 mg/ml. A cell suspension was adjusted to a density of 5 × 10^5^ cells/mL and uniformly seeded into the upper chamber (200 ul), ensuring consistent cell seeding per well. The lower chamber for MOF-Fe-treated groups contained 700 µl of medium with corresponding MOF-Fe concentrations. In comparison, the control group's lower chamber was supplemented with an equal volume of MOF-free medium. The upper chamber medium was serum-free, whereas the lower chamber contained a serum concentration of 20% to induce cell migration. The plate was then incubated at 37°C and 5% CO_2_ for 24 hours. Post-experiment, non-migratory cells in the upper chamber were removed, and cells traversed the membrane were fixed in 4% formaldehyde for 15 to 30 minutes. Subsequently, sections were stained in crystal violet solution for 20 to 30 minutes, then washed with PBS to remove excess stain. Finally, cells that migrated through the membrane were observed and counted under a microscope, thus assessing the impact of different concentrations of MOF on the migratory capability of RAW 264.7.

*Influence of MOF-Fe Particle Concentrations on ROS Alterations in RAW Cells*

This study aimed to assess the impact of MOF-Fe on ROS production in RAW 264.7 macrophage cells through experiments. The experimental design was divided into eight groups: control group without MOF, MOF-0.125, MOF-0.25, MOF-0.5, and control group + 10 µM H_2_O_2_, MOF-0.125 + 10 µM H_2_O_2_, MOF-0.25 + 10 µM H_2_O_2_, MOF-0.5 + 10 µM H_2_O_2_. RAW 264.7 cells were seeded in 6-well plates and then treated for 24 hours with complete medium and MOF at concentrations of 0.125, 0.25, and 0.5. Ten µM H_2_O_2_ was added to the MOF treatment for co-cultivation for one hour for the hydrogen peroxide-stimulated groups. Post-experiment, cells were stained using 2', 7'-dichlorofluorescein diacetate (DCFH-DA) as a ROS probe. Subsequently, the fluorescence intensity generated by each group of cells was observed using a fluorescence microscope to reflect the intracellular ROS levels.

*Influence of MOF-Fe Particle Concentrations on Glutathione Variations in RAW 264.7 Cells*

This study evaluated the influence of MOF-Fe on Glutathione (GSH) levels within RAW 264.7 macrophage cells. The experimental framework was divided into four groups: a control without MOF and three groups treated with MOF at concentrations of 0.125, 0.25, and 0.5 mg/ml. During the study, cells were cultured in 6-well plates and exposed to varying MOF solution concentrations. The duration of treatment spanned seven days, after which cells were lysed through three cycles of liquid nitrogen and a 37°C water bath. Subsequently, supernatants were collected following centrifugation at 12000 rpm for 10 minutes, and GSH concentrations were determined using a GSH assay kit (solarbio).

By comparing the GSH levels across different groups, the impact of MOF treatment on the antioxidative defence system within RAW 264.7 cells was assessed. As GSH is a principal intracellular antioxidant, its variations reflect the cellular response to oxidative stress, providing data support to understand the potential modulatory role of MOF-Fe in the oxidative stress status of macrophages.

*Impact of MOF-Fe Particle Concentrations on Differentiation of RAW 264.7 Cells Towards Osteoclasts*

This study designed a series of experiments to evaluate the impact of MOF-Fe on the differentiation of RAW 264.7 macrophage cells into osteoclasts. The experimental setup encompassed four groups: three MOF-treated cohorts at 0.125, 0.25, and 0.5 mg/ml concentrations and a control group without MOF. All experimental groups of RAW 264.7 cells were cultured in high-glucose DMEM containing 10% fetal bovine serum. Throughout the experiment, each group of cells was supplemented with medium enriched with Receptor Activator of Nuclear Factor Kappa-Β Ligand (RANKL, 50 ng/mL, novoprotein, China) and Macrophage Colony-Stimulating Factor (M-CSF, 30 ng/mL, novoprotein) to induce osteoclast differentiation. After seven days of culture, cellular samples were collected for total RNA extraction and reverse transcription to synthesise cDNA. Subsequently, the expression levels of specific genes were assessed using qPCR techniques, similar to previously described methods. The genes analysed, including beta-actin (as an internal reference gene), calreticulin (CALR), cathepsin K (CTSK), nuclear factor of activated T-cells, cytoplasmic 1 (NFATc1), osteoclast-associated receptor (OSCAR), and tartrate-resistant acid phosphatase 5 (TRAP5), are closely associated with osteoclast differentiation and function. By comparing the gene expression levels in cells across different treatment groups, the influence of MOF on the differentiation of RAW 264.7 cells towards osteoclasts was evaluated (**Supporting Information Table 2**).

**Supporting Information Table 2.** Primer sequences of RAW 264.7 used for quantitative PCR analysis (Mouse).

| Gene | Sequence forward (5'>3') | Sequence reverse (5'>3') |
| --- | --- | --- |
| ACTIN | GTCCCTCACCCTCCCAAAAG | GCTGCCTCAACACCTCAACCC |
| CTSK | TGTATAACGCCACGGCAAA | GGTTCACATTATCACGGTCACA |
| NFATc1 | GATGGCTCGCATGTTATTT | CTCACCACAGGGCTCACTA |
| OSCAR | CCGTGCTGACTTCACACCAA | GGGGTGACAAGGCCACTTTT |
| TRAP5 | TACCTGTGTGGACATGACC | CAGATCCATAGTGAAACCGC |
| CALR | TCAGGAACCACGGAATCCTC | ACATTCAAGCGGATGCGTCT |

*Promotive Effects of Different MOF-Fe Particles on Femoral Bone Repair in Rats*

Animal experiments and surgical operations were conducted in strict compliance with the guidelines of Shanghai Ninth People's Hospital (SYXK 2016-0016), rigorously following the ARRIVE guidelines, the UK Animals (Scientific Procedures) Act of 1986 and its subsequent iterations, and the regulations of Directive 2010/63/EU of the European Union.

To gauge the potential applications of MOF-Fe in rat bone repair, twenty-four 6-week-old male SD rats were selected and randomly apportioned into four parallel groups: control (n=6), MOF-0.125 (n=6), MOF-0.25 (n=6), and MOF-0.5 (n=6). Under 4% chloral hydrate anaesthesia and with pre-operative preparations meticulously administered, incisions were made in the hindlimb of each rat to unveil the femur. A subsequent bone defect, measuring 5 mm in length and 2 mm in width, penetrating to the marrow, was crafted in the midsection of the femur. Then, sterile saline and MOF suspensions of different concentrations were prepared, and after suturing the soft tissue, 1 ml was injected into the bone defects of the respective groups. The control group was administered 1 ml of saline. Post-surgical procedures permitted the rats unrestricted movement with no specific dietary constraints. Prophylactic antibiotics were routinely administered for three days post-surgery to thwart potential infections. On days 15 and 30 post-operation, bone tissue from each group underwent micro-computed tomography scans (SCANCO MEDICAL AG, Switzerland). Subsequently, 3D reconstructions of the results were facilitated. For analytical purposes, tissue samples were embedded in paraffin, sectioned, and subjected to Hematoxylin and Eosin (H&E), Masson's Trichrome, and Perl's staining.

In this study, sections were stained using Sirius Red dye to assess the impact of MOF-Fe on the alignment and maturation of collagen fibres in rat femurs. Following fixation, decalcification, embedding, and sectioning of the femoral samples, the sections were stained in Sirius Red solution (solarbio), which specifically binds to collagen fibres. By staining and microscopically observing sections from two time points, 15 and 30 days, the effect of MOF treatment on the arrangement and maturity of collagen fibres in femoral bone tissue was evaluated.

To evaluate the effect of MOF-Fe on the expression of BMP2 in rat femur sections. The femoral sections underwent fixation, decalcification, and embedding and were sliced into thin sections. These were then dewaxed, rehydrated, and subjected to standard antigen retrieval. Immunohistochemical experiments were conducted with overnight incubation in specific antibodies against BMP2 to detect the expression and localisation of these proteins in femoral tissue. A 20-minute incubation with secondary antibodies followed this. Finally, the expression of BMP2 in different treatment groups was evaluated and compared through microscopic observation and image capture.

*Preparation of Hydrogel Scaffolds*

This study prepared cylindrical Gel-60 hydrogel scaffolds containing different concentrations of MOF-Fe particles and explored their therapeutic potential in a New Zealand white rabbit bone defect model. The experimental design included four groups: a control without MOF and three concentration groups with MOF-0.125, MOF-0.25, and MOF-0.5. Initially, MOF particles at respective concentrations were uniformly blended into the Gel-60 hydrogel (EngForLife, China). Subsequently, this mixture was poured into custom-designed moulds to fabricate cylindrical scaffolds with 8 mm diameter and 3 mm height.

*Preparation of Circular Cranial Defect Model in New Zealand White Rabbits*

In this study, six New Zealand White rabbits were utilised as an animal model to evaluate the efficacy of different treatments in bone tissue repair. Before the experiments, the rabbits' selection, housing, and handling adhered to international animal ethics and welfare guidelines, maximising animal welfare and minimising unnecessary discomfort. Before commencement, this study obtained approval from the Animal Ethics Committee of Shanghai Ninth People's Hospital (SYXK2020-0025) and strictly followed all guidelines and regulations stipulated by the committee. Following the preparation of Gel60-MOF scaffolds, this study established a bone defect model in New Zealand White rabbits. Specifically, the rabbit skulls surgically created a circular cranial defect with a diameter of 8mm. Subsequently, the prepared hydrogel scaffolds from different treatment groups were implanted into the respective bone defects. All surgeries were conducted under strict aseptic conditions to reduce the risk of infection.

Post-implantation, the rabbits were allowed to recover and grow under standard conditions for 30 days, during which their health and the healing of the implantation sites were monitored. After 30 days, the rabbits were euthanised, and the cranial site containing the implanted scaffolds was extracted for further analysis.

*Statistical Analysis*

All data are presented as mean ± standard deviation or percentages. Independent sample t-tests were employed when comparing two sets of data. For samples of three or more, which conform to normal distribution and homogeneity of variance, one-way ANOVA was utilised. In contrast, the Kruskal-Wallis H test was adopted for data that did not meet these criteria. The threshold for significance was set at P < 0.05. Analyses were conducted using SPSS statistical software.


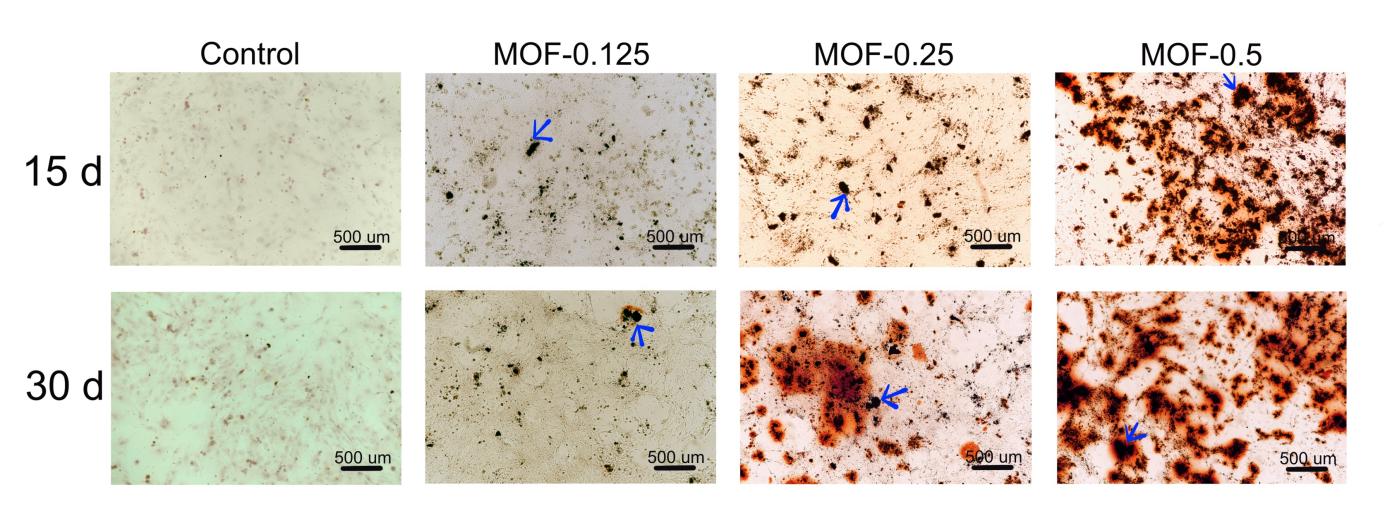
**Supporting Information Figure 1.** Image of bone marrow mesenchymal stem cells after Alizarin Red staining. Arrows point to iron-based metal-organic framework (MOF-Fe) particles, highlighting the increased mineral nodules around them.


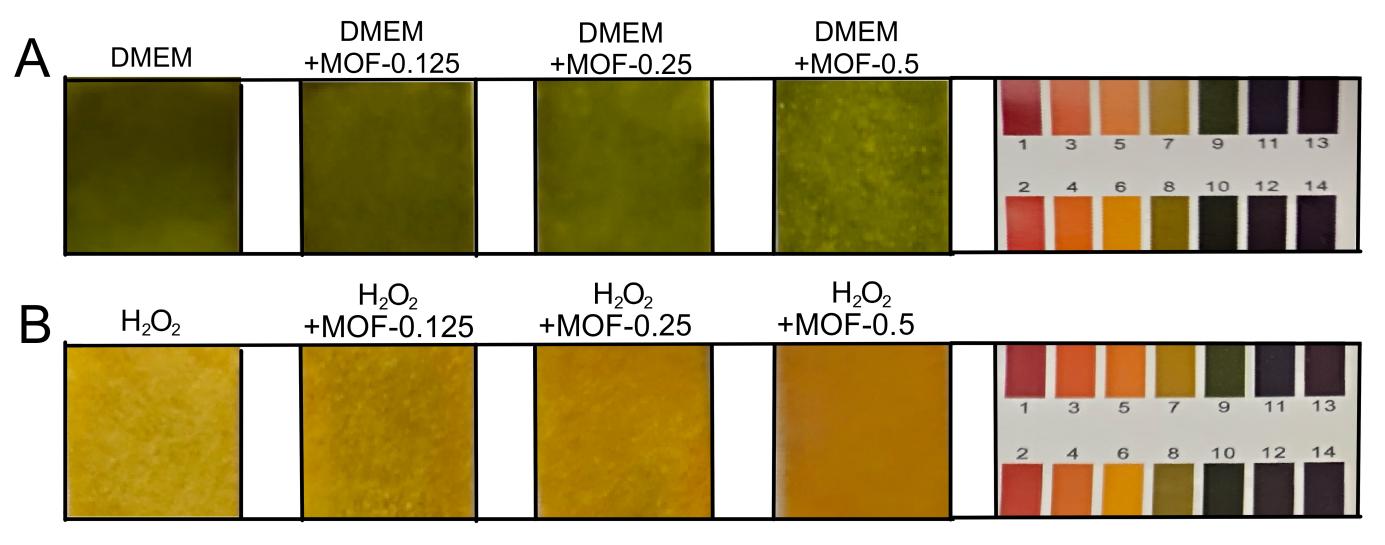


**Supporting Information Figure 2:** Effect of MOF-Fe Particle Addition on pH Values. (A) pH changes were detected using pH test strips after adding MOF-0.125, MOF-0.25, and MOF-0.5 to the complete culture medium, indicating that the pH range for all groups is within 7-9. (B) The pH range was detected using pH test strips when MOF-Fe catalysed the decomposition of hydrogen peroxide into oxygen and water.
