# Supplementary figures and images for: Biomimetic peroxidase MOF-Fe promotes bone defect repair by inhibiting TfR2 and activating the BMP2 pathway
Source: Biol Direct. 2024 Apr 23;19:30. doi: 10.1186/s13062-024-00473-2 (PMC11036606; doi:10.1186/s13062-024-00473-2)

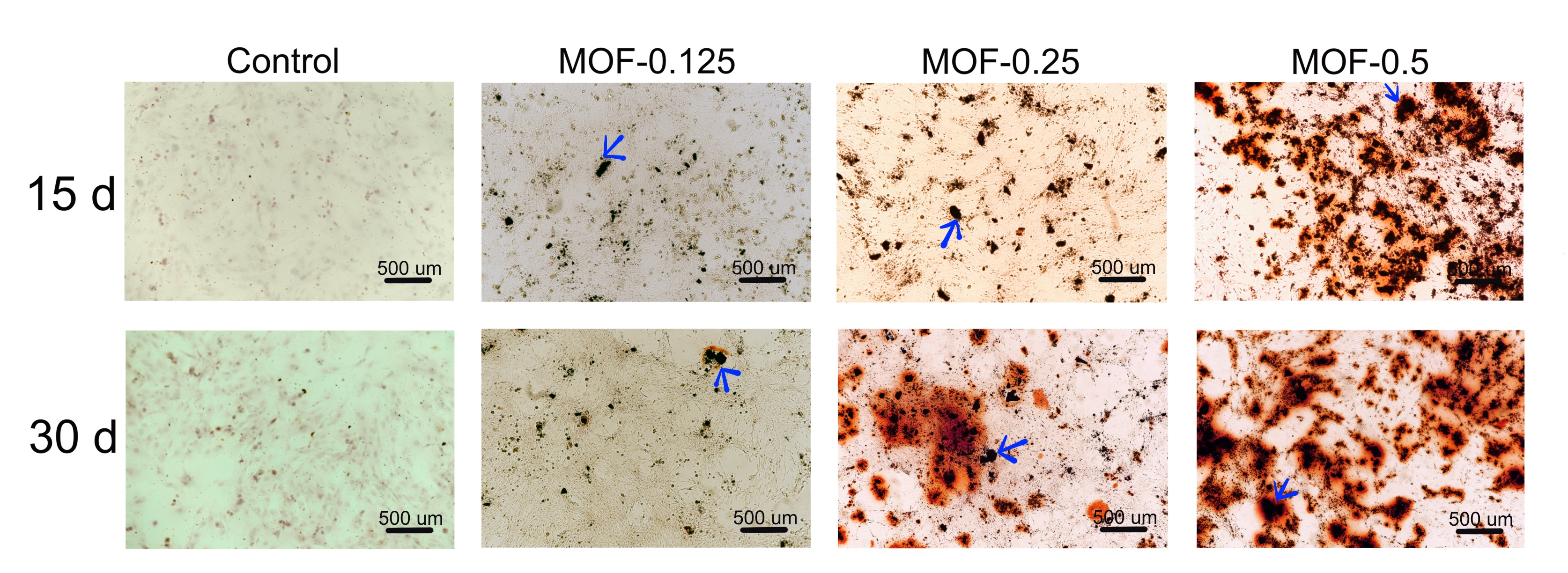

Supplement: Supplementary file 2 — Supplementary Material 2 [file 13062_2024_473_MOESM2_ESM.jpg]

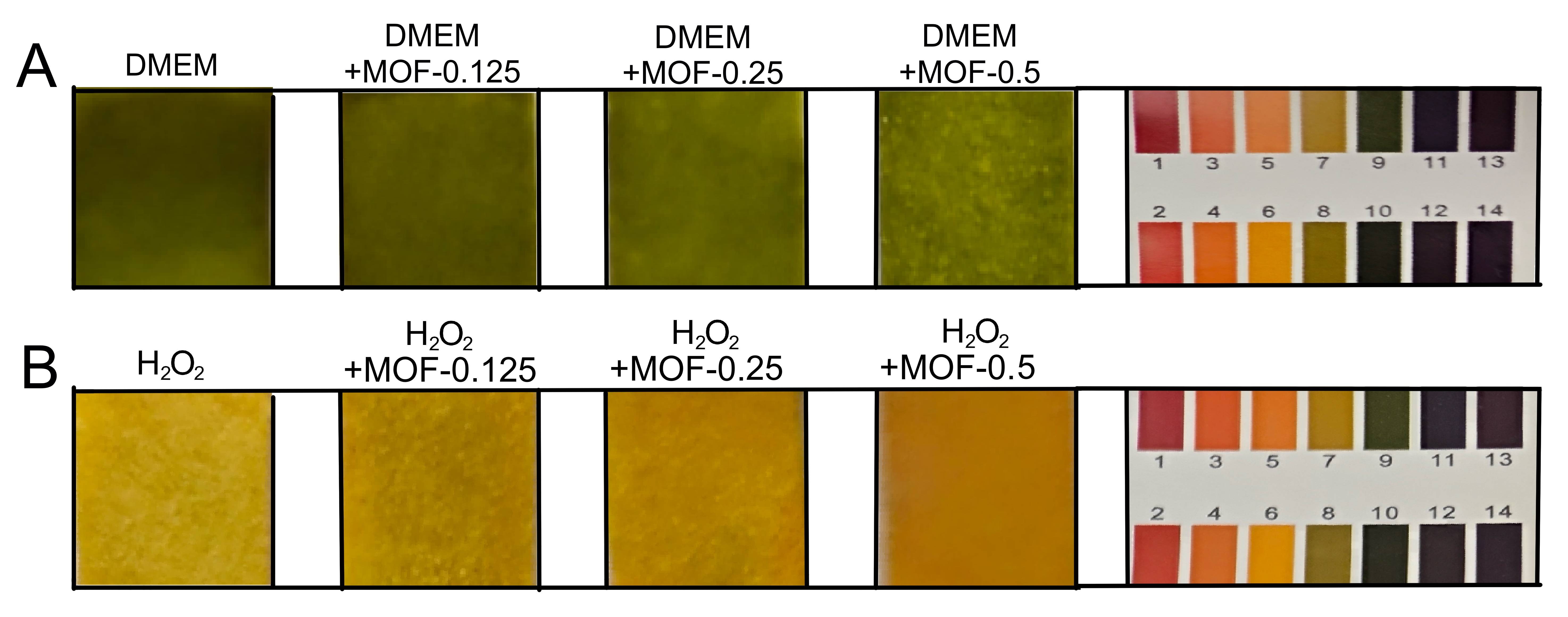

Supplement: Supplementary file 3 — Supplementary Material 3 [file 13062_2024_473_MOESM3_ESM.jpg]
